# Peroxiredoxin 1 promotes intestinal inflammation by activating the NLRP3 inflammasome in macrophages through lysosomal disruption in Crohn’s disease

**DOI:** 10.1038/s41419-025-07898-1

**Published:** 2025-07-26

**Authors:** Shenglan Li, Qiuping Xia, Ying He, Wei Wu, Damu Tang, Zhenghao Deng, Zhijun Zeng, Sha Tu, Bo Chen, Lei Gu, Xinyi Yang, Yu Peng, Huixiang Yang, Zhangzhe Peng

**Affiliations:** 1 Department of Ultrasonic Imaging, Xiangya Hospital, Central South University, Changsha, Hunan China; 2 Department of Nephrology, Xiangya Hospital, Central South University, Changsha, Hunan China; 3 Department of Gastroenterology, Xiangya Hospital, Central South University, Changsha, Hunan China; 4 Organ Fibrosis Research Center, Central South University, Changsha, Hunan China; 5 Reproductive Medicine Center, Xiangya Hospital, Central South University, Changsha, Hunan China; 6 Department of Infectious Diseases, The Second Xiangya Hospital of Central South University, Changsha, Hunan China; 7 Department of Geratology Surgery, Xiangya Hospital, Central South University, Changsha, Hunan China; 8 Urological Cancer Center for Research and Innovation (UCCRI), St Joseph’s Hospital, Hamilton, ON Canada; 9 Department of Pathology, Xiangya Hospital, Central South University, Changsha, Hunan China

**Keywords:** Crohn's disease, Inflammasome, Mucosal immunology

## Abstract

Damage-associated molecular patterns (DAMPs) are a cause of Crohn’s disease (CD). Peroxiredoxin 1 (Prdx1), a newly identified DAMP, plays a critical role in organ injury with its potent proinflammatory properties. However, its specific role in CD remains unclear. Here, we identify serum Prdx1 as a DAMP involved in CD. Serum Prdx1 levels were significantly increased and positively correlated with the severity of intestinal inflammation in both CD patients and mice with experimental colitis. Genetic knockout of *Prdx1* or administration of a Prdx1-neutralizing antibody attenuated colitis in mice, as evidenced by restoration of the colonic epithelium, improved disease activity, and reduced colonic inflammation. These protective effects were impaired by introduction of recombinant Prdx1 (rPrdx1). Mechanistically, Prdx1 exacerbated intestinal inflammation by promoting macrophage infiltration and subsequent cytokine production. Depletion of macrophages abolished the rPrdx1-mediated exacerbation of colitis. Further, rPrdx1 was internalized by macrophages, leading to lysosomal disruption and subsequent activation of the NLRP3 inflammasome. Pharmacological inhibition of NLRP3 effectively abrogated rPrdx1-induced exacerbation of colitis. In conclusion, serum Prdx1 promotes intestinal inflammation in CD at least in part by activating the NLRP3 inflammasome through lysosomal disruption in macrophages. These findings highlight the pathogenic role of Prdx1 in CD and reveal therapeutic potential of managing CD via neutralization of circulating Prdx1.

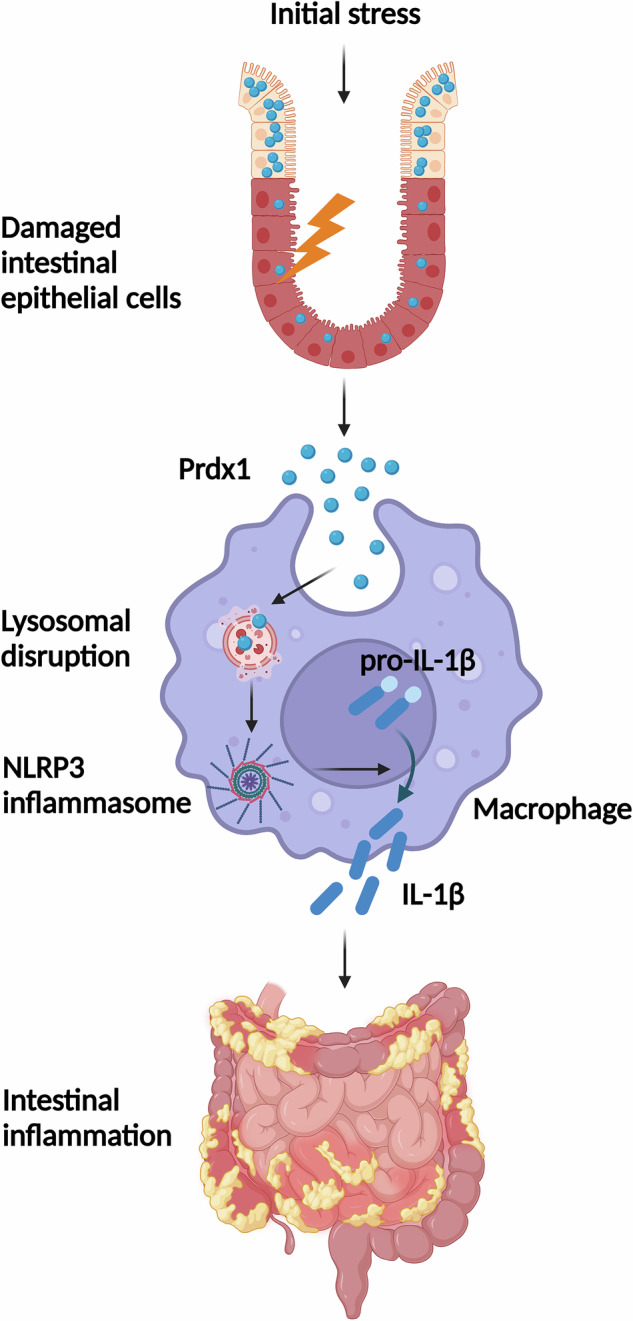

## Introduction

Crohn’s disease (CD), a clinical phenotype of inflammatory bowel disease (IBD), is a refractory intestinal inflammatory disorder that affects all regions of the gastrointestinal tract [1]. The prevalence of CD has been steadily rising in Western countries and is rapidly increasing in newly industrialized countries [2–4]. Abnormal and excessive inflammatory response in the intestinal mucosa is a characteristic feature of CD [5]. However, the molecular mechanism underlying abnormal intestinal mucosal inflammation in CD remains poorly understood.

Damage-associated molecular patterns (DAMPs) are distinct stimuli that initiate and maintain abnormal mucosal inflammation in CD. Damage to intestinal epithelial cells (IECs) leads to the release of DAMPs, which interact with pattern recognition receptors (PRRs), including Toll-­like receptors, nucleotide oligomerization domain­-like receptors and inflammasomes. This interaction activates the innate immune system and drives the subsequent intestinal inflammatory response [6]. In CD, the extensively inflamed intestinal mucosa serves as a concentrated reservoir of both local and systemic DAMPs [6], including high-mobility group box-1 (HMGB1) [7, 8], spliceosome-associated protein 130 (SAP130) [6], and mitochondrial DNA (mtDNA) [9]. Understanding the specific contributions of individual DAMPs to CD is critical to identify intervention targets.

Peroxiredoxin 1 (Prdx1) is a typical 2-Cys antioxidant protein that belongs to the peroxiredoxin family. It is commonly expressed in the cytosol, where it helps maintain cellular redox balance by reducing peroxide levels [10, 11]. Once released into the extracellular space, Prdx1 acts as a DAMP, promoting inflammation by binding to multiple PRRs. Emerging evidence has highlighted the crucial role of extracellular Prdx1 in triggering inflammation during ischemic brain injury and septic shock [12, 13]. Notably, Prdx1 is rapidly released following cardiopulmonary bypass, promoting acute inflammation by binding to Toll­-like receptor 2/4 [14]. Our previous studies have identified circulating Prdx1 as a novel DAMP in acute kidney and liver injuries, exacerbating these conditions by promoting inflammation [15, 16]. However, the specific role of Prdx1 in CD remains uncertain.

We report here a significant elevation of circulating Prdx1 in a cohort of CD patients, with levels positively correlating with the severity of intestinal inflammation. A similar increase in circulating Prdx1 is also observed in mice with experimental colitis. Circulating Prdx1 exacerbates colitis in mice, whereas genetic knockout of *Prdx1* or utilization of a Prdx1-neutralizing antibody markedly attenuates the development of colitis. Circulating Prdx1 induces intestinal inflammation by activating the NLRP3 inflammasome through lysosomal disruption in macrophages. Collectively, our research supports circulating Prdx1 as a novel DAMP for CD and proposes a potential therapeutic approach for treating CD by reducing circulating Prdx1.

## Results

### Circulating Prdx1 is significantly elevated in CD patients and mice with experimental colitis

**Fig. 1:**
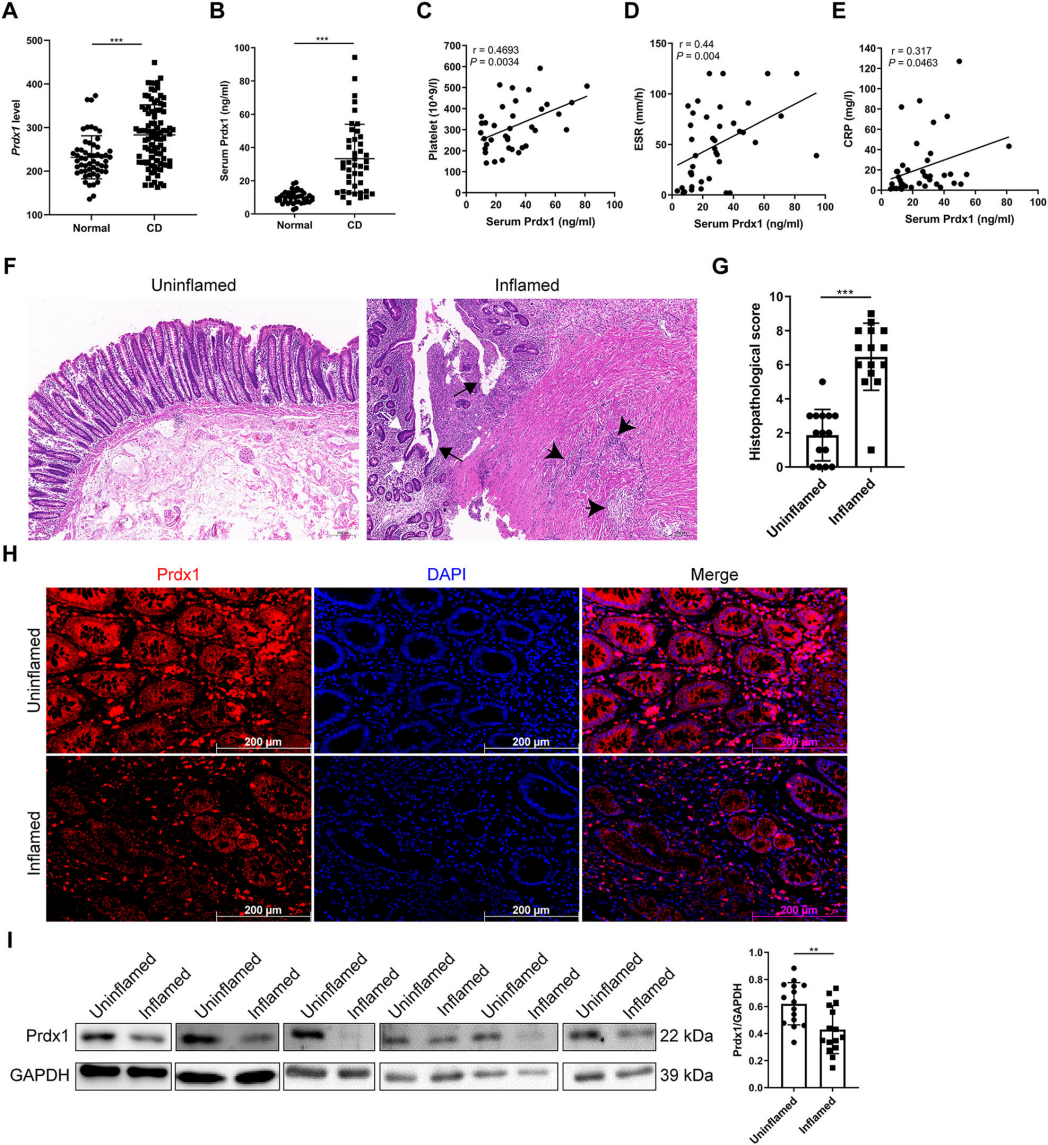
Circulating Prdx1 was elevated in patients with CD. **A** RNA-sequencing analysis of *Prdx1* mRNA expression in normal and CD samples obtained from GSE117993 (*n* = 55 for normal and *n* = 92 for CD). **B** Serum Prdx1 levels in normal participants and CD patients were measured by ELISA (*n* = 43 for each group). Serum Prdx1 was quantified with a standard ELISA curve. **C–E** The linear correlations between serum Prdx1 levels and platelet count (**C**), erythrocyte sedimentation rate (ESR) (**D**), and C-reactive protein (CRP) (**E**) in CD patients were analyzed by Pearson’s correlation coefficient. **F–G** Typical images of H&E staining and histopathological scores of uninflamed and inflamed colonic regions from the same CD patients (*n* = 15 for each group). Black arrow, ulceration with epithelial cell loss; white arrow, crypt deformation; black arrowhead, inflammatory cell infiltration. Scale bar, 50 μm. **H** Immunofluorescence staining for Prdx1 in the uninflamed and inflamed regions of colon from the same CD patients. Typical images are shown. Scale bar, 200 μm. **I** Western blot for Prdx1 expression in the uninflamed and inflamed regions of ileum/colon from the same CD patients (*n* = 15 for each group). Typical images and quantification are shown. Data are expressed as mean ± SD; ^**^P < 0.01, ^***^P < 0.001 by non-paired Student’s *t* test. Results presented were repeated at least three independent experiments for each experimental condition.

To investigate the role of Prdx1 in CD, we first analyzed the expression levels of Prdx1 in intestinal mucosal samples of CD patients from the Gene Expression Omnibus dataset (GSE117993). Data analysis revealed that *Prdx1* mRNA expression was significantly elevated in the mucosa of CD patients compared to that of normal participants (Fig. 1A). We then recruited a cohort consisting of normal participants and CD patients. Additional clinical characteristics are presented in Table 1. Compared with those in normal participants, significant increases in circulating Prdx1 were observed in CD patients (Fig. 1B). The levels of circulating Prdx1 were positively correlated with a set of inflammatory biomarkers, including platelet count (Fig. 1C), erythrocyte sedimentation rate (ESR; Fig. 1D), and C-reactive protein (CRP; Fig. 1E), suggesting a close association between circulating Prdx1 and the inflammatory activity of CD patients. We next examined the expression of Prdx1 in the inflamed and uninflamed regions of the ileum/colon of active CD patients who underwent enterectomy. Typical alterations were observed in the inflamed mucosa specimens, including crypt deformation, loss of epithelial cells, and infiltration of inflammatory cells in the lamina propria (Fig. 1F), accompanied by increased histopathological scores (Fig. 1G). Prdx1 was mainly expressed in the cytoplasm of IECs in the uninflamed region (Fig. 1H), consistent with previous findings [17]. However, significant reductions in Prdx1 were detected in IECs within inflamed tissues (Figs. 1H, I, and S1). Given the typical features of IEC death in the inflamed mucosa of CD patients, as well as the DAMP property of circulating Prdx1 released from damaged intrinsic cells [12, 15, 16], we hypothesized that elevated circulating Prdx1 in CD patients may originate from damaged IECs. Supporting this, we observed that tumor necrosis factor alpha (TNF-α) efficiently induced the release of Prdx1 into the extracellular space in HT29 and SW480 cells in a dose- and time-dependent manner (Fig. S2). Both HT29 and SW480 cells are colorectal cancer cell lines used as in vitro models for the intestinal epithelium because they retain many biochemical and physiologic features of the lower small intestine and ascending colon [18, 19]. These observations suggest that damaged IECs can release Prdx1 into the circulation, which results in increased serum Prdx1 and decreased mucosal Prdx1.

**Table 1:** Clinical characteristics of patients with active CD and normal participants.

Clinical Characteristics	Normal participants	CD
Number	43	43
Gender (Male/Female)	28/15	30/13
Age, y^a^	46.65 ± 13.28	31.5 ± 11.66
Disease duration, y^a^	-	2.99 ± 3.61
Origin of surgical specimens, n (%)	-	28 (65)
Ileum	-	16
Colon	-	12
Disease location, n (%)		
L1 (ileal)	-	18 (42)
L2 (colonic)	-	12 (28)
L3 (ileocolonic)	-	13 (30)
L4 (isolated upper disease)	-	0
Disease behavior, n (%)		
B1(non-stricturing, non-penetrating)	-	23 (53)
B2 (stricturing)	-	12 (28)
B3 (penetrating)	-	8 (19)
Perianal complications, n (%)	-	12 (28)
Smoking history, n (%)		
Never	32 (74)	38 (88)
Former	3 (7)	0
Current	8 (19)	5 (12)
Medication, n (%)		
Mesalazine	-	13 (30)
Corticosteroids	-	4 (9)
Immunosuppressants	-	6 (14)
Infliximab	-	5 (12)

*CD* Crohn’s disease.

^a^Data are expressed as mean ± SD.

**Fig. 2:**
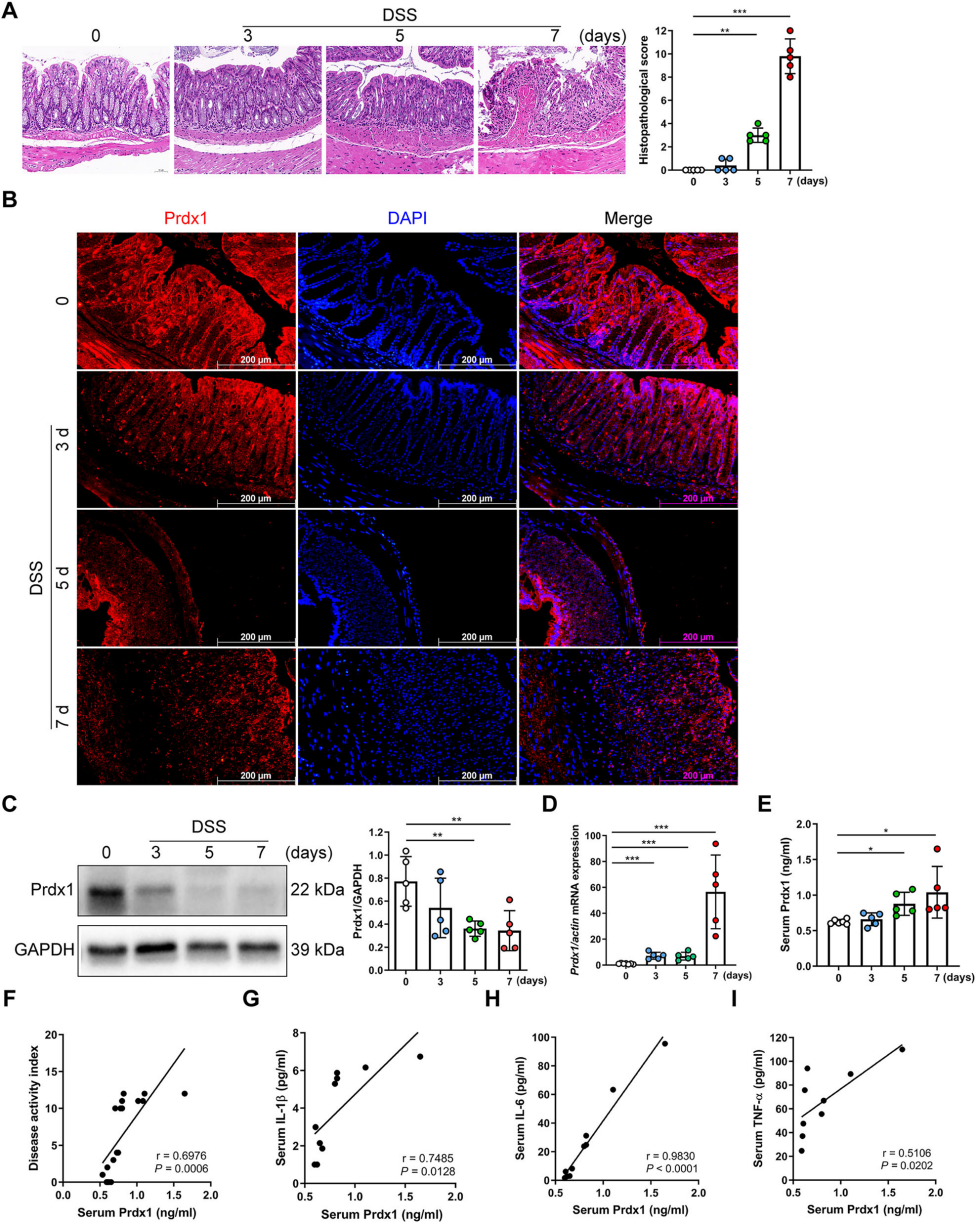
Serum Prdx1 was increased in mice with experimental colitis. Wild-type mice were treated with 4% DSS for the indicated time (*n* = 5 mice per group). **A** Typical images of H&E staining and histopathological scores of colon sections from control mice and those treated with DSS for varying durations. Scale bar, 50 μm. **B** Immunofluorescence staining for Prdx1 in colon sections from the indicated mouse groups. Typical images are shown. Scale bar, 200 μm. **C**, **D** *Prdx1* mRNA and protein expression in colon tissues from the indicated mouse groups were measured by RT-qPCR and western blot, respectively. Typical images and quantification are shown. **E** Serum Prdx1 levels in the indicated mouse groups were measured by ELISA and quantified using a standard curve. **F** The linear correlation between serum Prdx1 levels and disease activity index (DAI) scores in mice was analyzed by Pearson’s correlation coefficient. **G–I** The linear correlations between serum Prdx1 levels and levels of serum IL-1β, IL-6, and TNF-α were analyzed by Pearson’s correlation coefficient. Data are expressed as mean ± SD; ^*^P < 0.05, ^**^P < 0.01, ^***^P < 0.001 by non-paired Student’s *t* test. Results presented were repeated at least three independent experiments for each experimental condition.

To further verify these findings in vivo, we induced experimental colitis in mice using 4% dextran sulfate sodium (DSS). In the inflamed colons of mice with DSS-induced acute colitis, inflammatory cell infiltration appeared on Day 3 following DSS administration, with the injury worsening from Days 5 to 7, as evidenced by epithelial cell death, loss of crypt structure, and progression of transmural inflammation (Fig. 2A). These alterations are among the typical changes reported in DSS-induced acute colitis [20]. Similar to the findings observed in CD patients, Prdx1 was abundantly expressed in IECs of the colonic mucosa in control mice (Fig. 2B). However, in mice with DSS-induced acute colitis, Prdx1 expression in IECs progressively declined with increasing colonic injury (Fig. 2B, C). This reduction was unlikely due to decreases in gene expression, as *Prdx1* mRNA levels increased in the injured colon, peaking on Day 7 following DSS administration (Fig. 2D). The decrease of Prdx1 in IECs was accompanied by an increase of Prdx1 in the circulation of mice with acute colitis (Fig. 2E), further supporting the IECs origin of circulating Prdx1. The levels of serum Prdx1 positively correlated with the disease activity in mice with acute colitis (Fig. 2F). Furthermore, significant positive correlations were also observed between serum Prdx1 and key proinflammatory cytokines, including interleukin (IL)-1β, IL-6, and TNF-α, indicating an association between elevated serum Prdx1 and the severity of colonic inflammation (Fig. 2G–I). Overall, the elevation of circulating Prdx1 in both CD patients and mice with colitis strongly suggests its involvement in CD.

### Serum Prdx1 facilitates intestinal inflammation

**Fig. 3:**
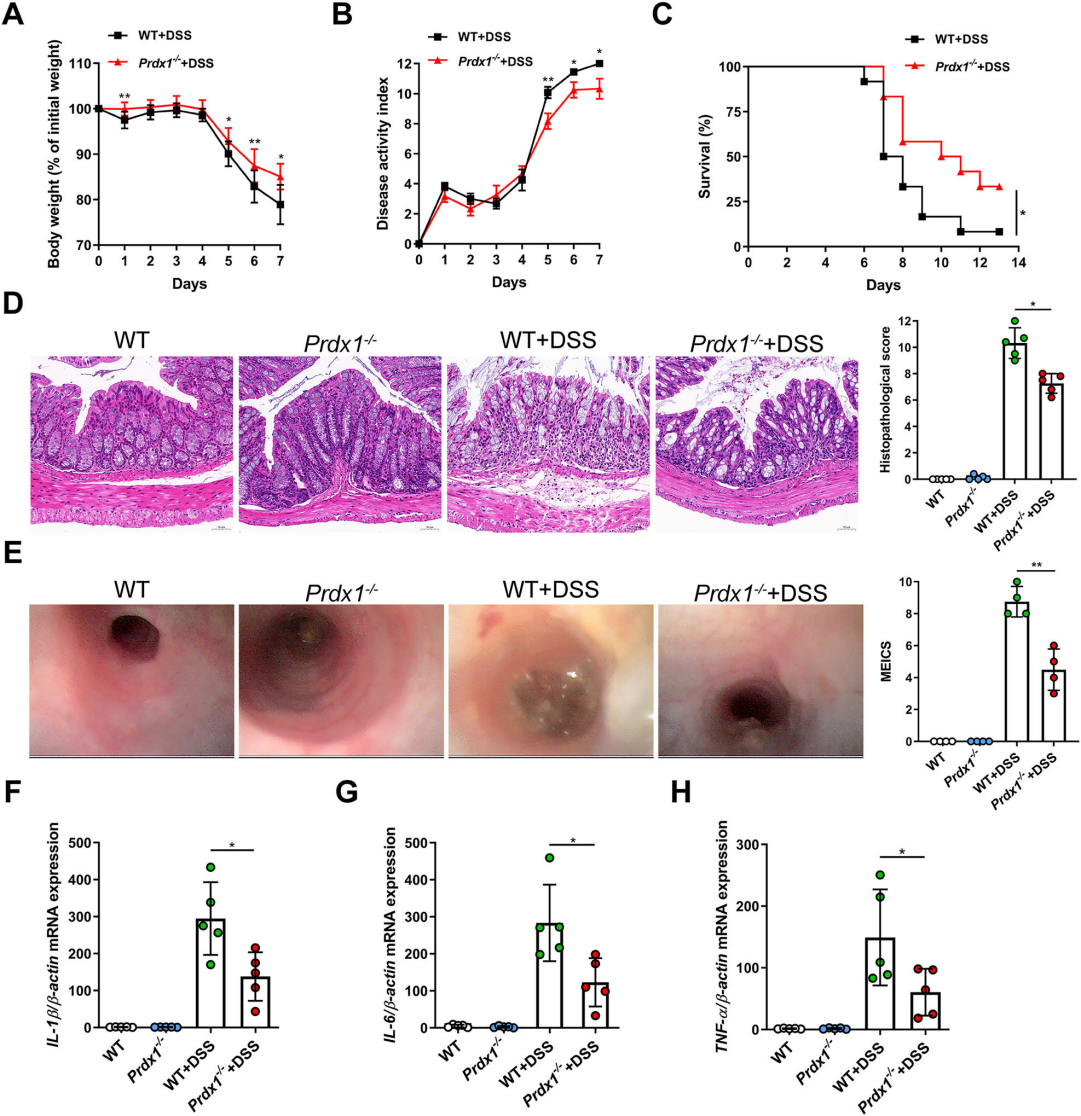
*Prdx1* knockout attenuated intestinal inflammation*.* *Prdx1*^*–/–*^ mice and their littermates received 4% DSS for 7 consecutive days. **A** Body weight of the mice was monitored daily and the data was plotted as a percentage of the initial body weight on Day 0 (*n* = 12 mice per group). **B** The disease activity of *Prdx1*^*–/–*^ mice and their littermates was monitored and scored daily (*n* = 12 mice per group). **C** Survival of mice monitored over a period of 14 days (*n* = 12 mice per group). **D** Typical images of H&E staining and histopathological scores of colon sections from the indicated mouse groups (*n* = 5 mice per group). Scale bar, 50 μm. **E** Colonic endoscopic images and murine endoscopic index of colitis severity (MEICS) scores of mice from the indicated groups (*n* = 4 mice per group). **F–H** The mRNA expression levels of *IL-1β*, *IL-6*, and *TNF-α* in the colons of indicated mouse groups were measured by RT-qPCR (*n* = 5 mice per group). Data are expressed as mean ± SD; ^*^P < 0.05, ^**^P < 0.01 by non-paired Student’s *t* test (**A–C**) or one-way ANOVA (**D–H**). Results presented were repeated at least three independent experiments for each experimental condition.

The detection of serum Prdx1 in CD patients and mice with colitis suggests that circulating Prdx1 is pathologically relevant to intestinal inflammation. To address the pathological contributions of circulating Prdx1 to intestinal inflammation, we examined the impact of Prdx1 depletion on colitis using *Prdx1* knockout (*Prdx1*^*–/–*^) mice (Fig. S3). Strikingly, *Prdx1*^*–/–*^ mice exhibited significant improvements in weight loss and disease activity index (DAI), which coincided with an increase in the survival rate (Fig. 3A–C). Consistent with these observations, *Prdx1*^*–/–*^ mice displayed reduced epithelial erosion, increased numbers of goblet cells, and decreased infiltration of inflammatory cells in both mucosal and submucosal layers, leading to lower histopathological scores for colonic injury and inflammation (Fig. 3D). Endoscopic analysis further confirmed reduced colonic injury and inflammation in *Prdx1*^*–/–*^ mice compared with their wild-type (WT) littermates, as evidenced by a more translucent and less granular colonic wall and no obvious signs of bleeding (Fig. 3E). Furthermore, Prdx1 deficiency significantly reduced the colonic expression of proinflammatory cytokines, including *IL-1β*, *IL-6*, and *TNF-α* (Fig. 3F–H).

**Fig. 4:**
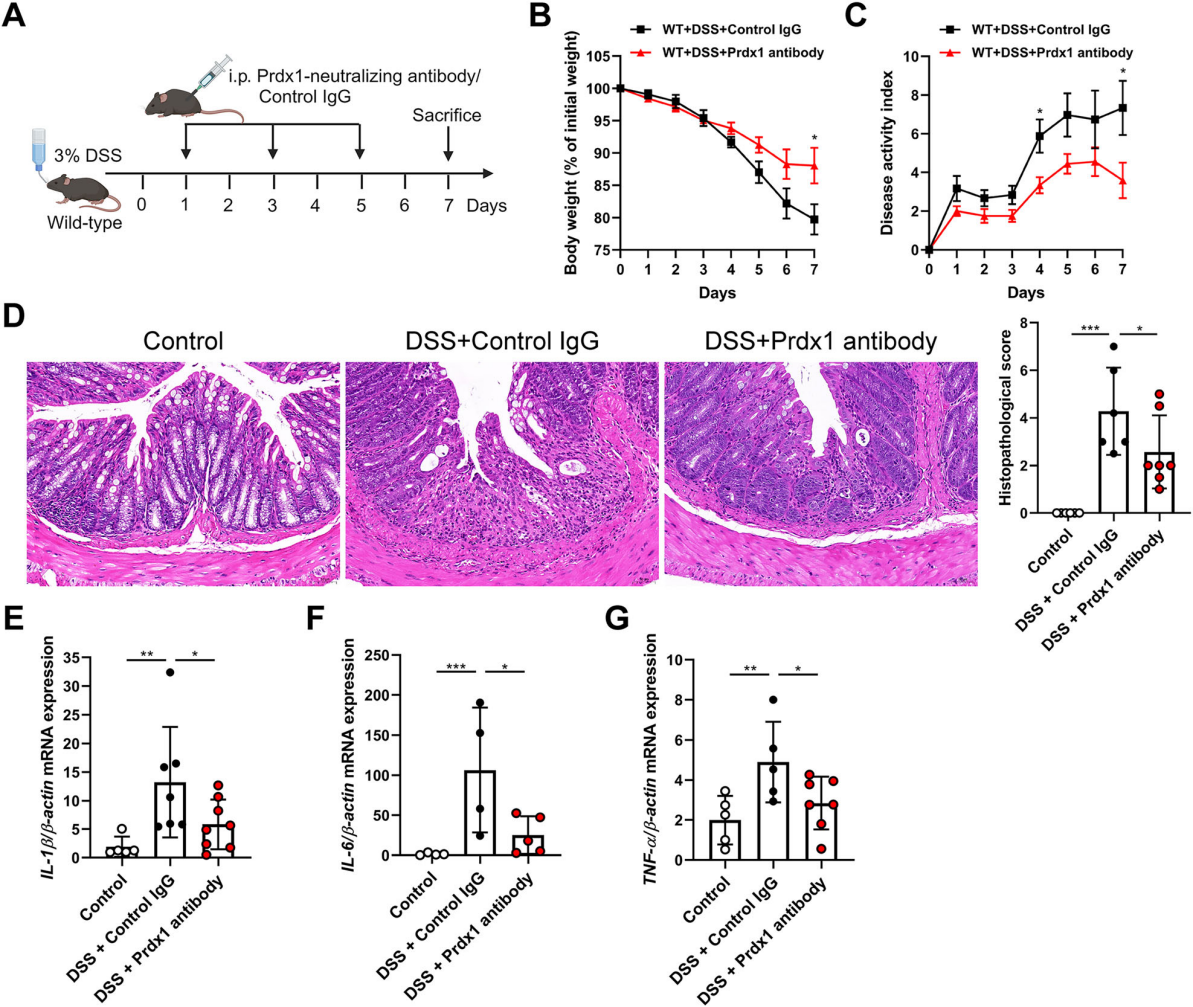
Prdx1 neutralization protected against intestinal inflammation. Wild-type mice were administered 3% DSS in their drinking water, followed by intraperitoneal (i.p.) injection of either control IgG or Prdx1-neutralizing antibody (150 μg/mouse) on Days 1, 3, and 5. The mice were sacrificed 7 days after DSS administration (*n* = 5–7 mice per group). **A** Schematic diagram of administration of Prdx1-neutralizing antibody to DSS-treated mice. **B** Body weight of the mice was monitored daily and the data was plotted as a percentage of the initial body weight on Day 0. **C** The disease activity was monitored and scored daily for each mouse. **D** Typical images of H&E staining and histopathological scores of colon sections from the indicated mouse groups. Scale bar, 50 μm. **E–G** The mRNA expression levels of *IL-1β*, *IL-6*, and *TNF-α* in the colons of indicated mouse groups were measured by RT-qPCR. Data are expressed as mean ± SD; ^*^P < 0.05, ^**^P < 0.01, ^***^P < 0.001 by non-paired Student’s *t* test (**B**, **C**) or one-way ANOVA (**D–G**). Results presented were repeated at least three independent experiments for each experimental condition.

To further validate this concept, we examined the effect of a Prdx1-neutralizing antibody on DSS-induced colitis (Fig. 4A). A preliminary safety assessment demonstrated a favorable safety profile for the antibody, with no detectable adverse effects observed (Fig. S4). Given the 50% mortality rate in WT mice 7 days after the administration of 4% DSS, we lowered the concentration of DSS to 3% in subsequent experiments. Compared with control IgG, the Prdx1-neutralizing antibody significantly alleviated DSS-induced colitis in WT mice, as indicated by increased body weight (Fig. 4B) and decreased disease activity (Fig. 4C), which were accompanied by decreased histopathological scores (Fig. 4D). Furthermore, the neutralizing antibody protected against DSS-induced intestinal inflammation, as evidenced by the significant decreases in colonic expression of *IL-1β*, *IL-6*, and *TNF-α* (Fig. 4E–G).

**Fig. 5:**
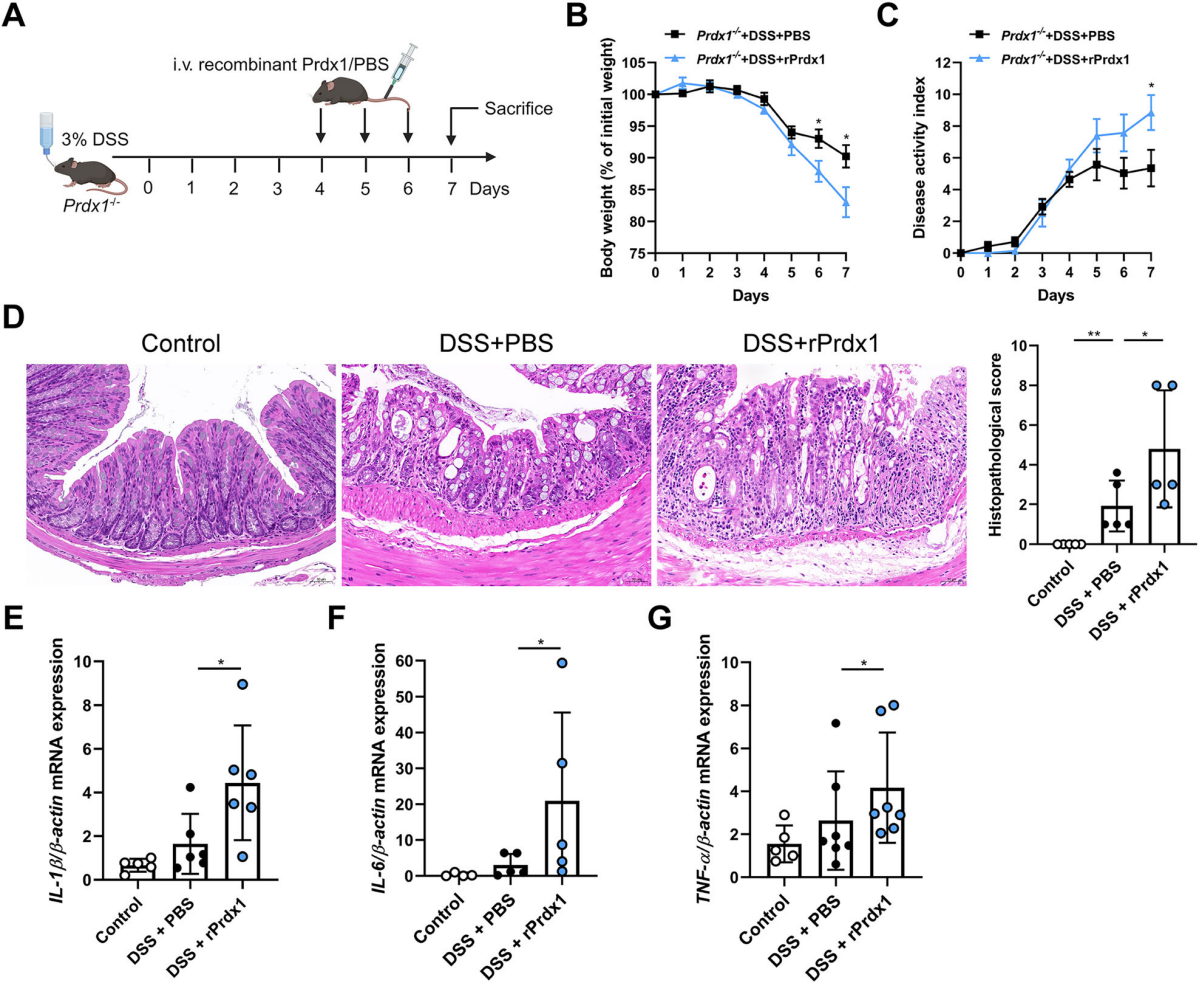
Reintroduction of recombinant Prdx1 (rPrdx1) facilitated intestinal inflammation. *Prdx1*^*–/–*^ mice and their littermates were administered 3% DSS in their drinking water, followed by intravenous (i.v.) injection of either phosphate-buffered saline (PBS) or rPrdx1 (10 μg/kg) on Days 4, 5, and 6. The mice were sacrificed 7 days after DSS administration (n = 5–7 mice per group). **A** Schematic diagram of administration of rPrdx1 to DSS-treated *Prdx1*^*–/–*^ mice. **B** Body weight of the mice was monitored daily and the data was plotted as a percentage of the initial body weight on Day 0. **C** The disease activity was monitored and scored daily for each mouse. **D** Typical images of H&E staining and histopathological scores of colon sections from the indicated mouse groups. Scale bar, 50 μm. **E–G** The mRNA expression levels of *IL-1β*, *IL-6*, and *TNF-α* in the colons of indicated mouse groups were measured by RT-qPCR. Data are expressed as mean ± SD; ^*^P < 0.05, ^**^P < 0.01 by non-paired Student’s *t* test (**B**, **C**) or one-way ANOVA (**D–G**). Results presented were repeated at least three independent experiments for each experimental condition.

To obtain additional evidence for a pathological role of circulating Prdx1 in colitis, we treated *Prdx1*^*–/–*^ mice with DSS along with intravenous (i.v.) reintroduction of recombinant Prdx1 (rPrdx1; Fig. 5A). Compared to vehicle-treated controls, rPrdx1 significantly increased the susceptibility of *Prdx1*^*–/–*^ mice to DSS-induced colonic injury and inflammation (Fig. 5B–D). Furthermore, marked elevations in colonic proinflammatory cytokines were observed in *Prdx1*^*–/–*^ mice reconstituted via i.v. injection of rPrdx1 in response to DSS treatment (Fig. 5E–G). Collectively, the above results demonstrate that circulating Prdx1 facilitates intestinal injury and inflammation.

### Serum Prdx1 induces intestinal inflammation by enhancing macrophage infiltration and interacting with macrophages

**Fig. 6:**
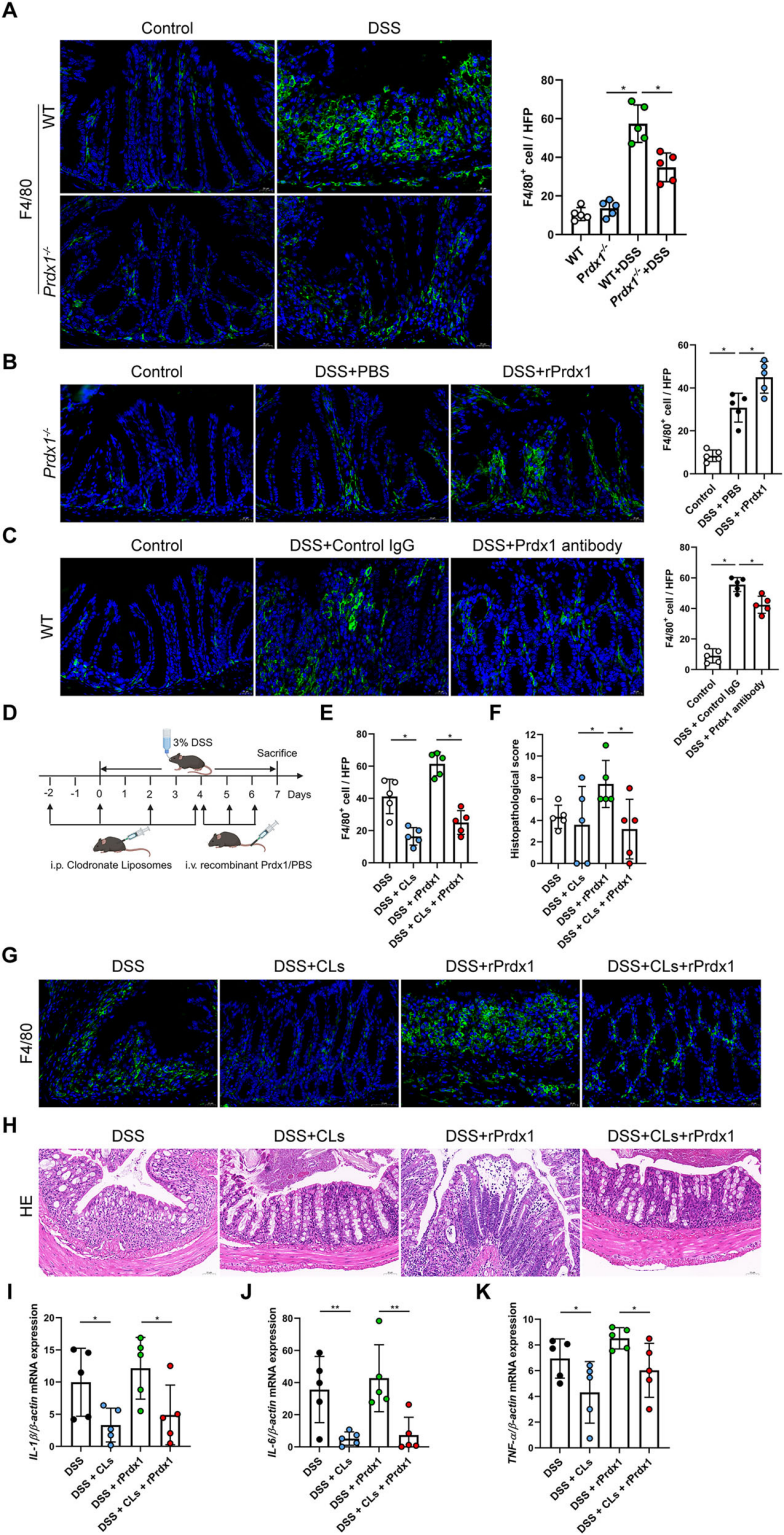
Serum Prdx1 induced intestinal inflammation by enhancing macrophage infiltration and interacting with macrophages. **A** Immunofluorescence staining for F4/80 in colon sections from *Prdx1*^*–/–*^ mice and their littermates treated with DSS (*n* = 5 mice per group). Typical images and quantification are included. Scale bar, 20 μm. **B** Immunofluorescence staining for F4/80 in colon sections from DSS-treated *Prdx1*^*–/–*^ mice along with intravenous (i.v.) injection of PBS or rPrdx1 (*n* = 5 mice per group). Typical images and quantification are included. Scale bar, 20 μm. **C** Immunofluorescence staining for F4/80 in colon sections from DSS-treated WT mice along with intraperitoneal (i.p.) injection of control IgG or Prdx1-neutralizing antibody (*n* = 5 mice per group). Typical images and quantification are included. Scale bar, 20 μm. **D–K** WT mice were depleted of macrophages using clodronate liposomes (CLs) and subsequently administered 3% DSS, accompanied by i.v. injection of either PBS or rPrdx1 (10 μg/kg; *n* = 5 mice per group). **D** Schematic diagram of DSS-treated mice with macrophage depletion. **E**, **G** Immunofluorescence staining for F4/80 in colon sections from the indicated mouse groups. Typical images and quantification are included. Scale bar, 20 μm. **F**, **H** Typical images of H&E staining and histopathological scores of colon sections from the indicated mouse groups. Scale bar, 50 μm. **I–K** The mRNA expression levels of *IL-1β*, *IL-6*, and *TNF-α* in the colons of indicated mouse groups were measured by RT-qPCR. Data are expressed as mean ± SD; *n* = 5 mice per group. ^*^P < 0.05, ^**^P < 0.01 by one-way ANOVA. Results presented were repeated at least three independent experiments for each experimental condition.

Macrophages are the key gatekeepers of intestinal immune homeostasis and play a crucial role in the pathogenesis of CD. As frontline cells of innate immunity, macrophages are responsible for the initial promotion and subsequent resolution of intestinal inflammation [21]. Upon recruitment to sites of injury, they activate inflammatory signaling pathways and drive the production of downstream proinflammatory cytokines. Given that circulating Prdx1 induces the production of colonic proinflammatory cytokines, we further examined its impact on macrophage infiltration in the colon. Following DSS administration, *Prdx1*^*–/–*^ mice exhibited significantly reduced infiltration of F4/80^+^ macrophages in the colonic mucosa compared to WT mice (Fig. 6A). Macrophage infiltration was attributed at least partially to circulating Prdx1, as i.v. injection of rPrdx1 remarkably increased mucosal macrophage infiltration in DSS-treated *Prdx1*^*–/–*^ mice (Fig. 6B), and neutralizing Prdx1 reduced the number of macrophages in the colonic mucosa of DSS-treated WT mice (Fig. 6C).

To further elucidate the pathogenic contributions of macrophages in Prdx1-mediated colitis, we depleted macrophages using clodronate liposomes (CLs; Fig. 6D). The application of CLs resulted in a 50% reduction in macrophage infiltration in the colon of DSS-treated mice (Fig. 6E, G). Consistent with findings in *Prdx1*^*–/–*^ mice, i.v. injection of rPrdx1 exacerbated DSS-induced colonic injury and inflammation in WT mice (Fig. 6F, H), accompanied by an increase in macrophage infiltration in the colon (Fig. 6E, G). Intriguingly, this exacerbation was abolished in mice with macrophage depletion (Fig. 6F, H). We further verified the role of macrophages in Prdx1-mediated intestinal inflammation by examining the expression of proinflammatory cytokines in the colon. Macrophage depletion by CLs significantly alleviated DSS-induced inflammation, as evidenced by reduced colonic levels of *IL-1β*, *IL-6*, and *TNF-α* (Fig. 6I–K). Moreover, the rPrdx1-induced elevation of these cytokines was markedly suppressed in the absence of macrophages (Fig. 6I–K). Collectively, these results indicate that macrophages are critical mediators of Prdx1-driven intestinal inflammation.

### Prdx1 activates the NLRP3 inflammasome in macrophages

**Fig. 7:**
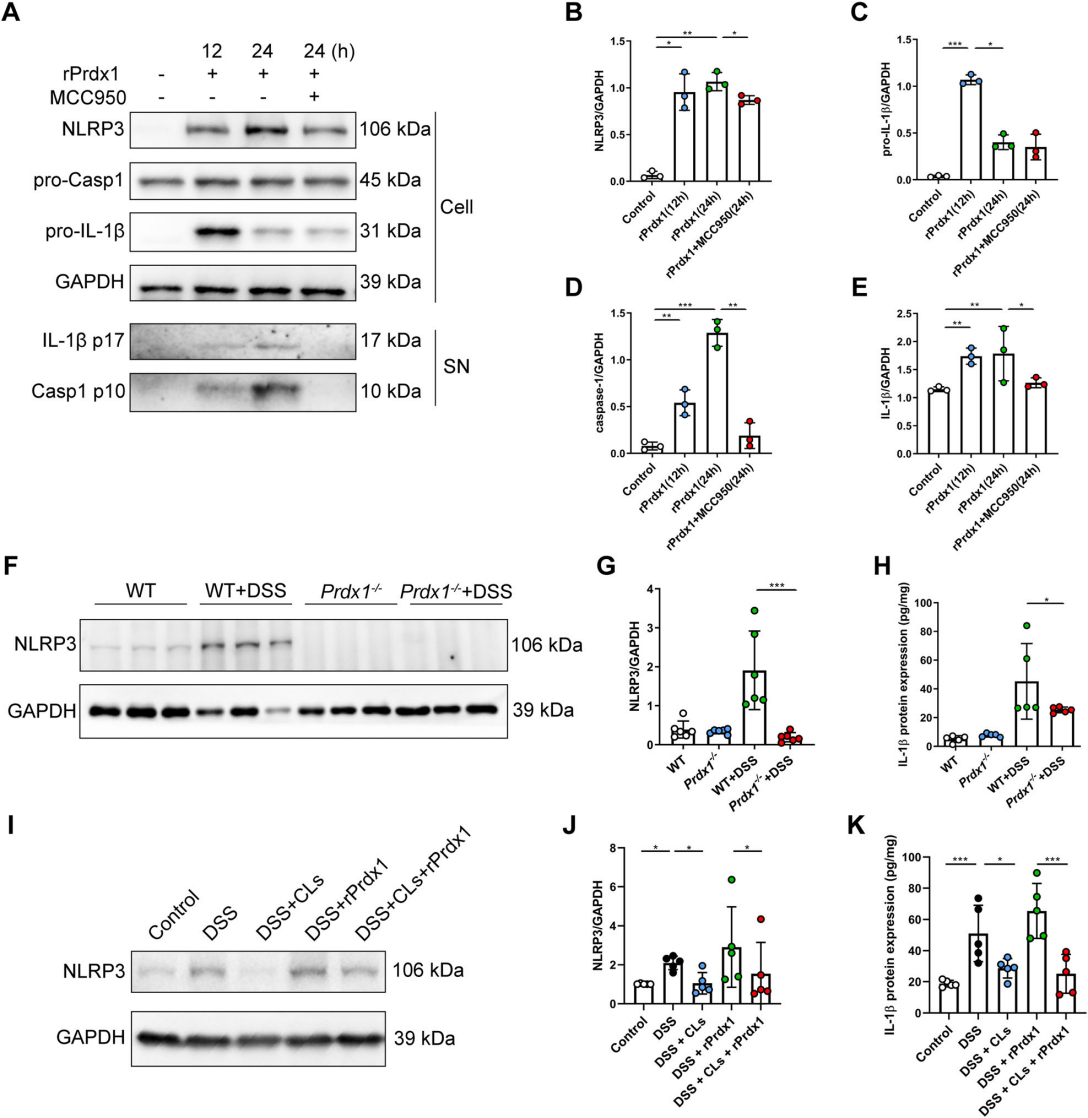
Prdx1 activates the NLRP3 inflammasome in macrophages. **A–E** Primary peritoneal macrophages (PPMs) isolated from wild-type mice were stimulated with rPrdx1 (25 nM) for 12 and 24 hours (h) with or without pretreatment of MCC950 (10 μM for 1 h). Cells were collected and analyzed for the expression levels of pro-caspase-1 (pro-Casp1) (**A**), NLRP3 (**B**), and pro-IL-1β (**C**). Conditioned medium was harvested and analyzed for cleaved Casp1 (**D**) and mature IL-1β (**E**) expression levels. Typical images and quantification are included (*n* = 3 biological repeats). **F**, **G** NLRP3 protein expression and quantification in colon tissues from *Prdx1*^*–/–*^ mice and their littermates treated with DSS (*n* = 6 mice per group). **H** IL-1β levels in colon tissues from the indicated mouse groups were detected by ELISA (*n* = 6 mice per group). **I–K** WT mice were depleted of macrophages using clodronate liposomes (CLs) and subsequently administered 3% DSS, accompanied by i.v. injection of either PBS or rPrdx1 (10 μg/kg; *n* = 5 mice per group). NLRP3 protein expression and quantification (**I**, **J**), along with IL-1β levels (**K**) were detected in colon tissues from the indicated mouse groups. Data are expressed as mean ± SD; ^*^P < 0.05, ^**^P < 0.01, ^***^P < 0.001 by one-way ANOVA. Results presented were repeated at least three independent experiments for each experimental condition.

We next sought to explore the signaling pathways in macrophages through which Prdx1 drives CD-associated intestinal inflammation. Given the well-established role of the NLRP3 inflammasome in modulating macrophage-mediated inflammatory responses in CD [22–25], we hypothesized that Prdx1 may exert its proinflammatory effects through activation of the NLRP3 inflammasome in macrophages. To investigate this, we isolated primary peritoneal macrophages (PPMs) from mice and examined the effect of Prdx1 on NLRP3 inflammasome activation. Surprisingly, rPrdx1 treatment notably upregulated the expression of NLRP3 and pro-IL-1β, and induced caspase-1 cleavage in PPMs (Fig. 7A–D). These events were accompanied by the production of mature IL-1β, which began at 12 hours (h) post-stimulation and continued to increase at 24 h (Fig. 7A, E). Importantly, pharmacological inhibition of NLRP3 inflammasome activation with MCC950 completely abolished rPrdx1-induced IL-1β production (Fig. 7A, E). These results indicate that Prdx1 activates the NLRP3 inflammasome in macrophages.

To further investigate the relationship between Prdx1 and NLRP3 inflammasome in intestinal inflammation, we examined the impact of Prdx1 depletion on the activation of the intestinal NLRP3 inflammasome. The expression of NLRP3 was significantly elevated in the colon tissues of WT mice treated with DSS; important to note is that this elevation was essentially suppressed in DSS-treated *Prdx1*^*–/–*^ mice (Fig. 7F, G). Consistently, IL-1β production in the colon was markedly lower in *Prdx1*^*–/–*^ mice than in WT mice following DSS treatment (Fig. 7H).

To validate that Prdx1 activates the NLRP3 inflammasome in macrophages during intestinal inflammation, we next evaluated the effect of macrophage depletion on this activation. Depletion of macrophages significantly reduced the DSS-induced upregulation of NLRP3 expression in colon tissues of WT mice (Fig. 7I, J). Intravenous administration of rPrdx1 increased colonic NLRP3 expression; intriguingly, this effect was greatly diminished in mice with macrophage depletion (Fig. 7I, J). Consistently, CLs treatment resulted in a marked decrease in IL-1β levels in colon tissues of DSS-treated mice (Fig. 7K). Notably, the rPrdx1-induced elevation of IL-1β levels was also effectively abolished in macrophage-depleted mice (Fig. 7K). Together, these results suggest that Prdx1 promotes intestinal inflammation at least in part by activating the NLRP3 inflammasome in macrophages.

### Cell-free Prdx1 induces lysosomal disruption to activate the NLRP3 inflammasome

**Fig. 8:**
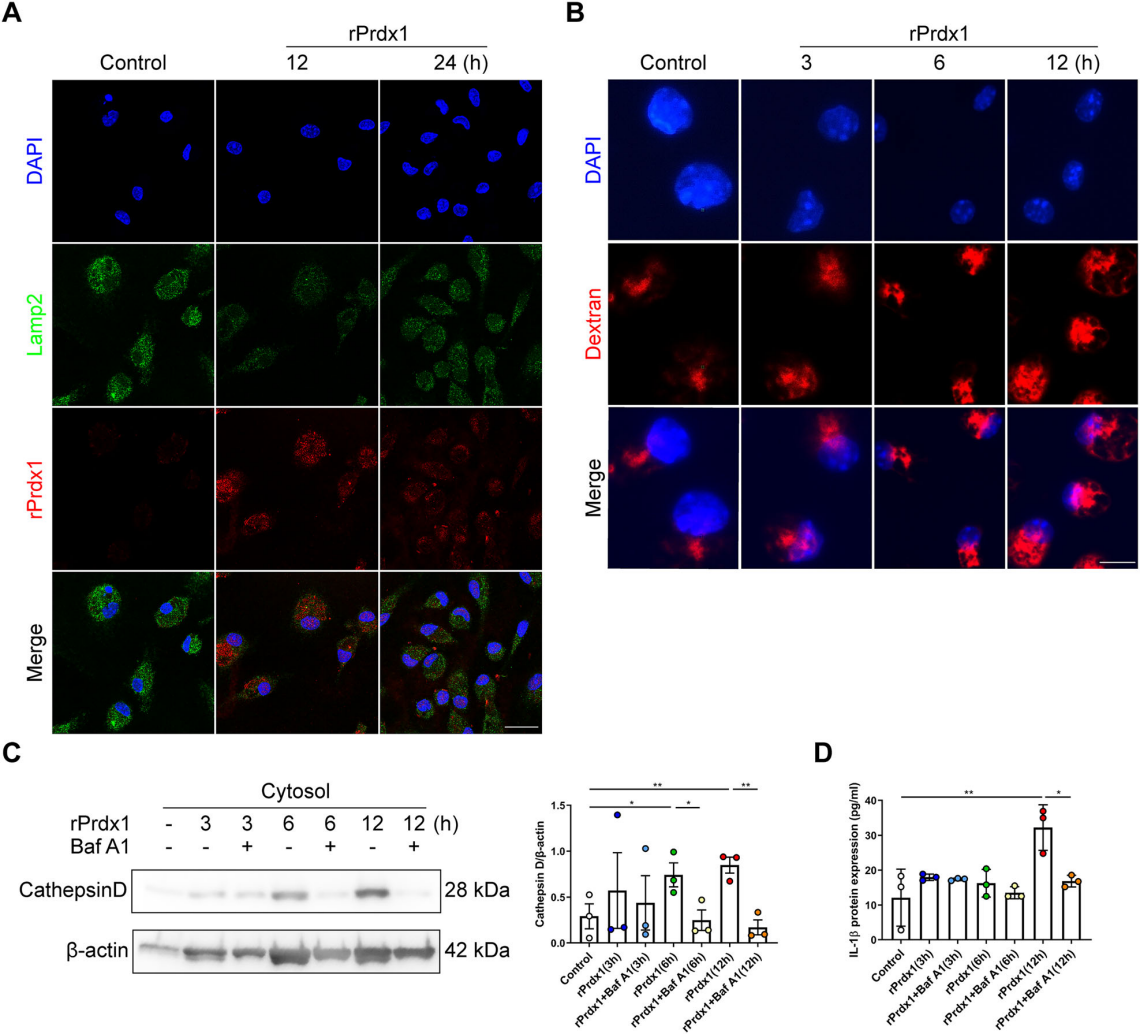
Cell-free Prdx1 induces lysosomal disruption to activate the NLRP3 inflammasome. **A** PPMs isolated from *Prdx1*^*–/–*^ mice were stimulated with rPrdx1 at 25 nM for 12 and 24 hours (h). Immunofluorescence staining was performed to detect Prdx1 (red) and Lamp2 (green) in PPMs, and typical images are shown. Scale bar, 20 μm. **B** PPMs from WT mice were preincubated with fluorescent dextran and then stimulated with rPrdx1 at 25 nM for 3, 6, or 12 h. Immunofluorescence staining for dextran was conducted, and typical images are shown. Scale bar, 10 μm. **C**, **D** PPMs from WT mice were stimulated with rPrdx1 (25 nM) for 3, 6, or 12 h, with or without pretreatment of bafilomycin A1 (Baf A1, 100 nM for 1 h). **C** The levels of cathepsin D in the cytosolic fraction were measured and quantified. **D** Conditioned medium was harvested and analyzed for IL-1β levels by ELISA. Data are expressed as mean ± SD; ^*^P < 0.05, ^**^P < 0.01, ^***^P < 0.001 by one-way ANOVA. Results presented were repeated at least three independent experiments for each experimental condition.

We next further explored the mechanisms underlying Prdx1-mediated activation of the NLRP3 inflammasome. The mechanisms of NLRP3 inflammasome activation, as supported by most studies, include the efflux of potassium (K^+^), lysosomal disruption, mitochondrial dysfunction, and other factors [26, 27]. It has been reported that Prdx1 can be selectively internalized by F4/80^+^ infiltrating mononuclear phagocytes for lysosomal degradation, indicating a potential link between Prdx1 and lysosomes [28]. To clarify this relationship, we incubated PPMs derived from *Prdx1*^*–/–*^ mice with rPrdx1 and performed co-labeling of Prdx1 and lysosomes. Immunofluorescence staining for LAMP2, a lysosomal membrane marker, revealed the typical punctate lysosomal pattern under basal conditions (Fig. 8A). However, following 12 and 24 h of rPrdx1 treatment, the LAMP2 signal became diffuse and exhibited a marked reduction in fluorescence intensity, indicative of lysosomal disruption (Fig. 8A). At the same time, a significant intracellular Prdx1 signal was detected (Fig. 8A), suggesting that extracellular Prdx1 had been internalized by the macrophages. To further assess the role of internalized Prdx1, PPMs from WT mice were loaded with dextran, a fluorescent dye that accumulates in lysosomes. Immunofluorescence revealed that exposure to rPrdx1 led to the transfer of dextran from lysosomes to the cytosol in a time-dependent manner, which was further evident after 12 h of rPrdx1 stimulation (Fig. 8B). Similar results were obtained when utilizing DQ-ovalbumin, a different fluorescent dye that remains inactive unless proteolytically processed into peptides in lysosomes (Fig. S5A). These results suggest that cell-free Prdx1 can be internalized by macrophages and induces lysosomal rupture.

To further confirm that Prdx1 induces lysosomal rupture and the subsequent release of lysosomal contents into the cytosol, we isolated the cytosol devoid of cytoplasmic membranes, endosomes, and lysosomes by utilizing low concentrations of digitonin (Fig. S5B) [29] and measured the amount of cathepsin D, a lysosomal protease. Notably, exposure to rPrdx1 resulted in the detection of cathepsin D in the cytoplasmic compartment in a time-dependent manner, further indicating lysosomal rupture in response to rPrdx1 (Fig. 8C). Lysosomal acidification, preceding lysosomal swelling and damage, is essential for NLRP3 inflammasome activation [26]. Treatment with bafilomycin A1, a lysosomal proton pump inhibitor that alkalizes the lysosome, robustly inhibited the release of cathepsin D triggered by rPrdx1 in PPMs (Fig. 8C). Consistently, the secretion of IL-1β was increased in the supernatant of PPMs after 12 h of rPrdx1 stimulation, which was remarkably inhibited by co-treatment with bafilomycin A1 (Fig. 8D), indicating that inhibiting lysosomal rupture effectively abolishes Prdx1-mediated NLRP3 inflammasome activation. Moreover, we observed minimal effects of cell-free Prdx1 on other established mechanisms involved in NLRP3 inflammasome activation, including K^+^ efflux and mitochondrial reactive oxygen species (mtROS) production (Fig. S5C, D). Taken together, these observations show that cell-free Prdx1 induces lysosomal rupture, thereby activating the NLRP3 inflammasome.

### NLRP3 inflammasome plays a critical role in facilitating Prdx1-mediated colitis

**Fig. 9:**
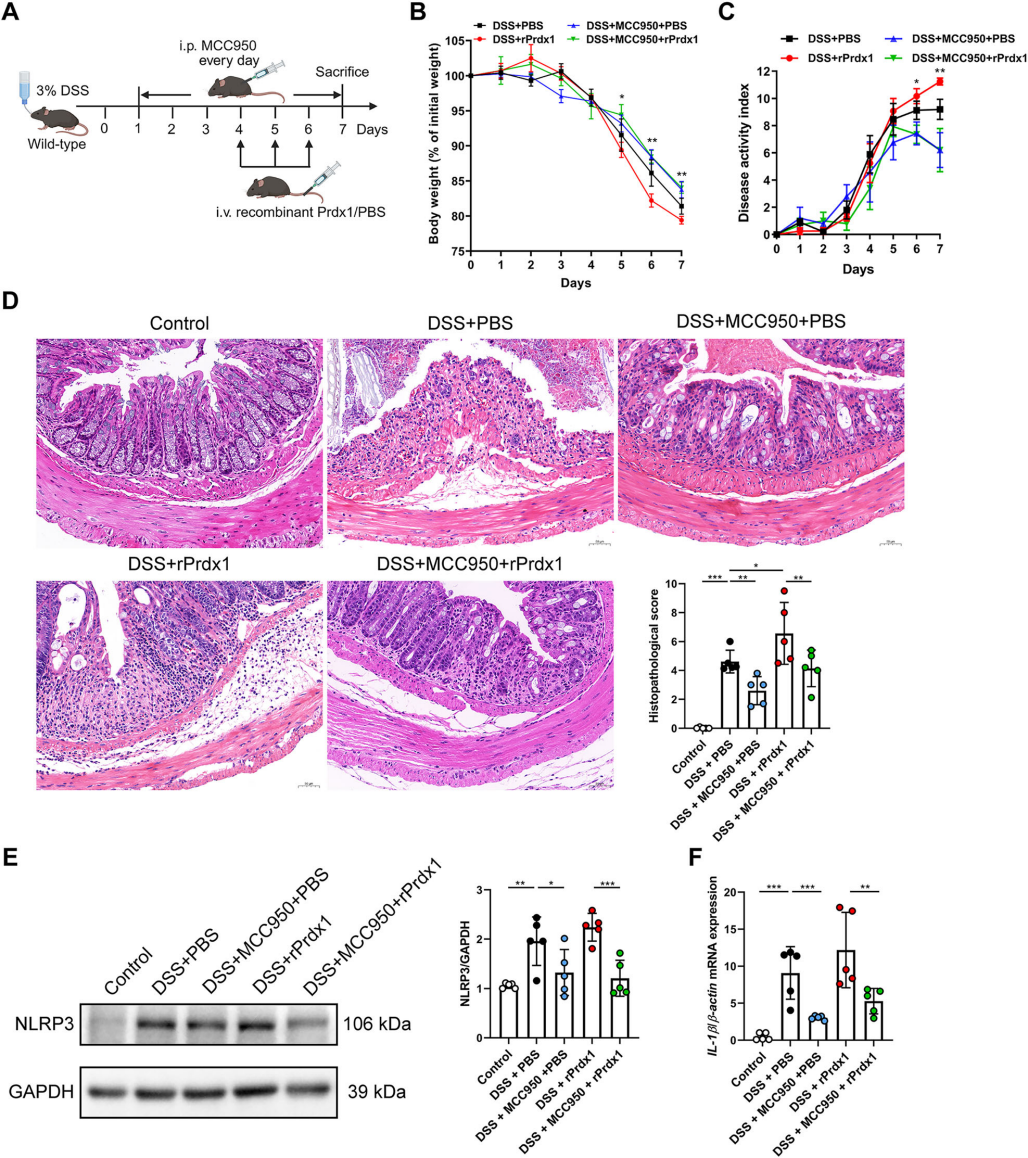
NLRP3 inflammasome plays a critical role in facilitating Prdx1-mediated colitis. Wild-type mice were administered 3% DSS for 7 consecutive days, accompanied by intraperitoneal (i.p.) injection of either PBS or MCC950 (20 mg/kg) every day. Mice were then intravenously (i.v.) injected with rPrdx1 on Days 4, 5, and 6 (10 μg/kg; *n* = 5 mice per group). **A** Schematic diagram of administration of MCC950 to DSS-treated mice. **B** Body weight of the mice was monitored daily, and the data was plotted as a percentage of the initial body weight on Day 0. **C** The disease activity was monitored and scored daily for each mouse. **D** Typical images of H&E staining and histopathological scores of colon sections from the indicated mouse groups. Scale bar, 50 μm. **E**, **F** NLRP3 protein expression and quantification (**E**), along with IL-1β levels (**F**), were detected in colon tissues from the indicated mouse groups. Data are expressed as mean ± SD; ^*^P < 0.05, ^**^P < 0.01, ^***^P < 0.001 by one-way ANOVA. Results presented were repeated at least three independent experiments for each experimental condition.

To further elucidate the pathological contributions of the NLRP3 inflammasome in Prdx1-mediated colitis, we employed the NLRP3 inhibitor MCC950 in a DSS-induced acute colitis model (Fig. 9A). MCC950 treatment alleviated DSS-induced weight loss, DAI, and colonic tissue injury (Fig. 9B, C). As expected, intravenous administration of rPrdx1 exacerbated DSS-induced colitis, as demonstrated by further reductions in body weight and increased DAI scores (Fig. 9B, C). Importantly, this exacerbation was effectively reversed by MCC950 treatment (Fig. 9B, C). Histopathological analysis further confirmed that MCC950 significantly attenuated rPrdx1-mediated colonic injury and inflammation (Fig. 9D). We next examined the effect of MCC950 on Prdx1-mediated activation of the intestinal NLRP3 inflammasome. As anticipated, MCC950 decreased NLRP3 expression in colon tissues of mice with acute colitis and notably suppressed the upregulation induced by rPrdx1 (Fig. 9E). Correspondingly, IL-1β production was markedly reduced in the colon of rPrdx1-treated colitis mice following MCC950 administration (Fig. 9F). Collectively, these results provide strong in vivo evidence that Prdx1 exacerbates colonic injury and inflammation through activation of the NLRP3 inflammasome.

## Discussion

**Fig. 10:**
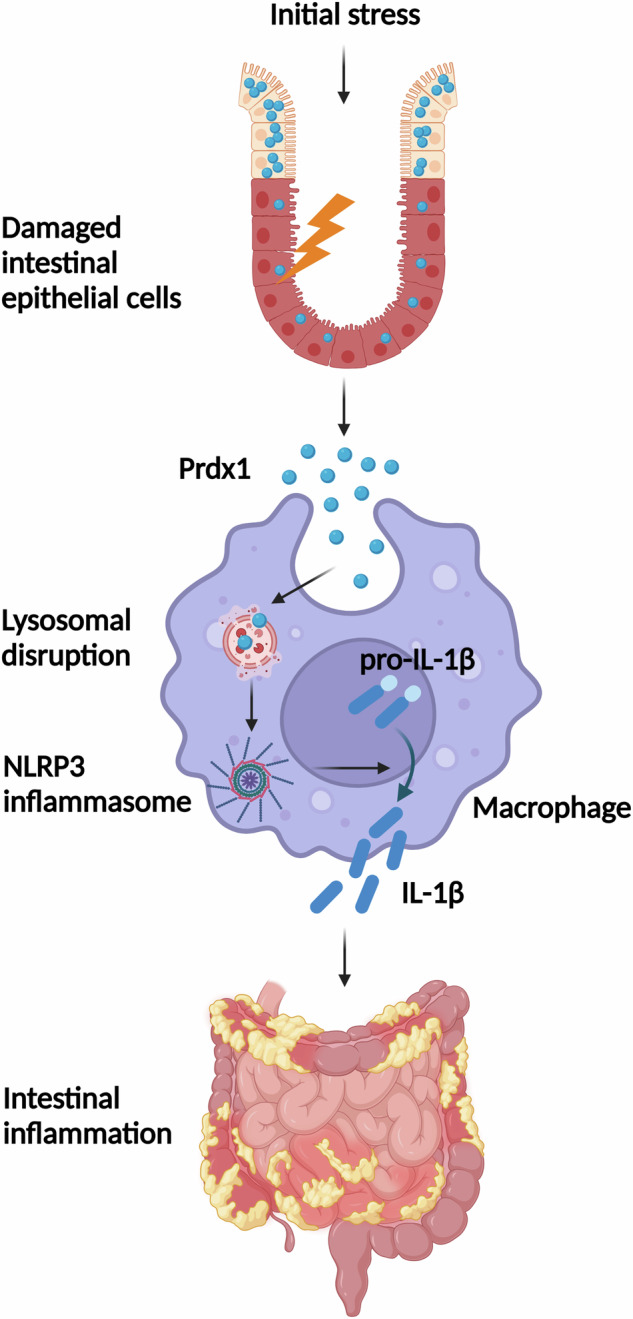
Schematic diagram illustrates cell-free Prdx1 as a critical damage-associated molecular pattern for promoting intestinal inflammation in CD. Initial stress to the intestinal epithelium triggers the release of Prdx1, which induces macrophage infiltration and is subsequently internalized by macrophages, leading to lysosomal disruption and activation of the NLRP3 inflammasome. This cascade results in the production of inflammatory cytokines, ultimately contributing to intestinal inflammation in CD.

In CD, stress or damage to the intestinal epithelium causes the release of DAMPs, which are important pathogenic stimuli activating immune cells and maintaining abnormal mucosal inflammation [6]. In this study, we specifically explored the elevation of circulating Prdx1 in CD patients and mice with experimental colitis. Our findings demonstrate the pathological role of circulating Prdx1 as a novel DAMP in facilitating intestinal inflammation through the induction of macrophage infiltration and activation of the NLRP3 inflammasome, followed by the production of inflammatory cytokines. Collectively, these processes contribute to the pathogenesis and progression of CD (Fig. 10).

Few studies have examined the relationship between Prdx1 and intestinal inflammation. Our findings indicate that serum Prdx1 contributes to intestinal inflammation. This is supported by the reduced severity of colitis observed in *Prdx1*^*–/–*^ mice and in mice treated with a Prdx1-neutralizing antibody. Moreover, this protective effect was reversed upon administration of rPrdx1 to *Prdx1*^*–/–*^ mice. The pathogenic role of Prdx1 in colitis aligns with findings in irritable bowel syndrome (IBS), where elevated serum levels of Prdx1 have been associated with disease progression [17]. Although IBS and CD differ essentially, both are characterized by dysregulation of the innate immune system. However, the protective effects conferred by lowering circulating Prdx1 remained incomplete, indicating the involvement of other factors in CD. These factors may include other DAMPs associated with CD, such as HMGB1 [7, 8], SAP130 [6], and mtDNA [9]. Additionally, other Prdxs may also be involved in intestinal inflammation, as evidenced by the protective effects observed in *Prdx6*-deficient mice in models of acute and chronic colitis [30]. Nonetheless, our results clearly identify serum Prdx1 as a significant DAMP contributing to the pathogenesis of CD.

Currently, a range of monoclonal antibodies have been introduced for the treatment of CD, including anti-TNF, anti-integrin, and anti-IL-12/23 agents. However, most CD patients fail to achieve sustained remission with these therapies, which are often associated with high rates of failure and intolerance [31]. This underscores the urgent need to identify novel therapeutic targets. Given their role as key initiators of inflammation, DAMPs represent a particularly novel and promising class of therapeutic targets in CD. In our study, the application of a monoclonal antibody targeting Prdx1 significantly improved disease outcomes in DSS-induced colitis mice, including restoration of colonic epithelial integrity, preservation of mucosal architecture, decrease in disease activity, and attenuation of colonic inflammation. These findings highlight Prdx1 as a promising therapeutic target, potentially opening new avenues for treatment in CD patients who are resistant to conventional therapies.

The mechanisms by which Prdx1 promotes intestinal inflammation are not completely understood. Here, we demonstrated that Prdx1 plays a pivotal role in regulating the inflammatory response through the induction of macrophage infiltration and subsequent cytokine production during disease progression. Although macrophage depletion did not significantly improve colonic histopathology in mice with colitis, consistent with previous findings [32], it effectively abolished the exacerbation of colitis induced by rPrdx1 administration. This further supports the critical role of macrophages in Prdx1-mediated intestinal inflammation. Nonetheless, the interactions of Prdx1 with other inflammatory cells in intestinal inflammation merit further investigation.

The NLRP3 inflammasome plays a critical role in modulating inflammatory responses in CD. Pharmacological inhibition of the NLRP3 inflammasome pathway has been shown to decrease IL-1β production and mitigate colonic inflammation [25, 33]. In this study, we provide evidence that extracellular Prdx1 activates the NLRP3 inflammasome in the intestine: (1) Induction of NLRP3 and IL-1β is significantly attenuated in the colon of *Prdx1*^*–/–*^ mice compared to WT mice following DSS challenge; (2) rPrdx1 activates the NLRP3 inflammasome in PPMs; (3) rPrdx1 is internalized by PPMs, resulting in lysosomal disruption and subsequent activation of the NLRP3 inflammasome; and (4) pharmacological inhibition of NLRP3 with MCC950 effectively abrogated rPrdx1-induced exacerbation of colitis and the associated upregulation of colonic NLRP3 and IL-1β expression. These findings demonstrate a functional link between extracellular Prdx1 and NLRP3 inflammasome in intestinal inflammation. However, it has been reported that the curcumin analogue AI-44 drives the binding of intracellular Prdx1 to pro-caspase-1, thereby suppressing NLRP3 inflammasome activation in macrophages. Notably, this binding appears to be pharmacologically induced, as their data indicated that Prdx1 does not naturally bind pro-caspase-1 under unstimulated conditions [34]. These observations imply that intracellular and extracellular Prdx1 may play distinct roles in the regulation of the inflammasome.

In conclusion, the current study demonstrates that serum Prdx1 acts as a novel DAMP in CD and promotes intestinal inflammation by activating the NLRP3 inflammasome through lysosomal disruption in macrophages. These findings advance the understanding of CD pathogenesis and provide potential clinical applications for managing CD via neutralization of circulating Prdx1.

## Methods and materials

### Human participants

Clinical protocols were approved by the Medical Ethics Committee of Xiangya Hospital, Central South University (Approval No. 201803490). Serum samples were collected from active CD patients and healthy donors from Xiangya Hospital, Central South University. Surgical specimens were obtained from the inflamed ileum/colon and adjacent uninflamed ileum/colon of CD patients who underwent enterectomy. All CD patients were diagnosed according to the ECCO-ESGAR Guideline for Diagnostic Assessment in IBD [35, 36]. Exclusion criteria included severe systematic diseases such as autoimmune diseases, tumors, hematological or infectious diseases, pregnancy or lactation, inability or unwillingness to provide informed consent, or individuals with other diseases affecting the intestines. The healthy donors were invited meeting the criteria that aged 14 to 70 and without any systematic diseases or drug history.

### Mouse models

Male C57BL/6 J mice (8–10 weeks old) were purchased from GemPharmatech Co., Ltd. (Jiangsu, China). Male *Prdx1*^*–/–*^ mice and their littermates on a C57BL/6 J background were generated in our laboratory using the CRISPR/Cas9 technique. All mice used in this study were kept at the Department of Laboratory Animals of Central South University under specific pathogen-free conditions. All animal experimental protocols and procedures were approved by the Ethics Review Committee for Animal Experimentation of Central South University. All animal experiments were conducted and analyzed in a blinded manner.

A DSS-induced acute colitis mouse model was established by administering 4% DSS (MW 36,000–50,000, MP Biomedicals, California, USA) in drinking water for 7 consecutive days [20]. DSS solution was replaced every 2 days. The body weight and DAI were monitored daily. The score was calculated based on the following parameters: weight loss (0: none, 1: 1−5%, 2: 6−10%, 3: 11−18%, 4: >18%); stool consistency (0: normal, 2: loose stool, 4: diarrhea); and fecal blood content (0−1: normal, 2: positive hemoccult, 4: gross rectal bleeding) [20]. Mice were sacrificed 3, 5 or 7 days after DSS administration.

For the neutralization of Prdx1, WT mice received 3% DSS for 7 days, along with intraperitoneal injection of either control IgG or monoclonal Prdx1-neutralizing antibody (150 μg/mouse) on Days 1, 3, and 5. For preliminary safety assessment of the Prdx1-neutralizing antibody, healthy WT mice received the antibody according to the same dosing and administration schedule used in the acute colitis model, with monitoring over a 14-day period.

For the reintroduction of rPrdx1, *Prdx1*^*–/–*^ mice were administered 3% DSS in their drinking water for 7 consecutive days, along with intravenous injection of either phosphate-buffered saline (PBS) or rPrdx1 (10 μg/kg) on Days 4, 5, and 6.

For a macrophage depletion model, WT mice were intraperitoneally injected (200 μL per mouse) with either CLs (#40337ES08, Yeasen, Shanghai, China) or control liposomes (#40338ES05, Yeasen) 2 days before exposure to DSS and at Days 0, 2, and 4 during DSS treatment. The effectiveness of macrophage depletion was confirmed by examining the number of colon-resident macrophages by immunofluorescence staining for F4/80.

To inhibit NLRP3 inflammasome activation in vivo, WT mice received daily intraperitoneal injections of MCC950 (20 mg/kg) from days 0 to 7 during DSS administration, whereas control groups were administered PBS.

### Materials

A human Prdx1 enzyme-linked immunosorbent assay (ELISA) kit (#KA0536, Abnova, Taipei City, China), mouse ELISA kits for Prdx1 (#CSB-EL018653MO, CUSABIO, Wuhan, China), IL-1β (#88-7013-88, ThermoFisher Scientific, Massachusetts, USA), IL-6 (#88-7064-22, ThermoFisher Scientific), and TNF-α (#88-7324-22, ThermoFisher Scientific) were used. Monoclonal Prdx1-neutralizing antibody and control IgG were obtained from Abmart (Shanghai, China). Full-length rPrdx1 (#P4543, Abnova) was generated using an Escherichia coli expression system with a purity greater than 95% (http://www.abnova.com). His-rPrdx1 (#WG-03145D) was generated from ABclonal (Wuhan, China). MCC950 (#S7809) and bafilomycin A1 (#S1413) were obtained from Selleckchem (Texas, USA). The antibodies were detailed in Table S1.

### Endoscopy and scoring

Mice were anesthetized with isoflurane and subjected to volume-controlled ventilation with positive end-expiratory pressure. The mice were then subjected to colonic irrigation with saline repeatedly to remove the stools. The endoscope was carefully inserted into the rectum and pushed as far as possible under visual control. The recording started when the endoscope was slowly withdrawn. The endoscopic score was determined according to the murine endoscopic index of colitis severity (MEICS) system [37].

### Histology and immunofluorescence staining

The formalin-fixed colon tissues were embedded in paraffin and cut into 4 μm sections for hematoxylin and eosin (H&E) staining. Epithelium was scored as: 0: normal morphology; 1: loss of goblet cells; 2: loss of crypts (10–50%); 3: loss of crypts in large areas (50-90%); 4: totally loss of crypts with incomplete epithelium; 5: small to medium sized ulcers (less than 10 crypts width); 6: large ulcers (more than 10 crypts width). Infiltration was scored at 3 levels: 1) mucosa was scored as: 0: no infiltrate; 1: mild infiltrate (inflammatory cell infiltration < 1/3); 2: moderate infiltrate (inflammatory cell infiltration < 2/3); 3: severe infiltrate (inflammatory cell infiltration > 2/3, infiltrate reaching the L. muscularis mucosa); 2) submucosa was scored as: 0: no infiltrate; 1: mild to moderate infiltrate; 2: severe infiltrate; 3) muscularis/serosal layer was scored as: 0: no infiltrate; 1: mild to severe infiltrate. Each of the 5 anatomical segments was scored. The total histopathological score represents the sum of the epithelium and infiltration scores, and thus ranges from 0 to 12.

The paraffin slices were incubated at 65 °C for 1 h, then dewaxed and rehydrated through gradient ethanol and washed in PBS. After retrieval by pepsin, the slices were washed with PBS for 3 times and then blocked by goat serum for 1 h at room temperature. The slices were then treated with primary antibodies overnight at 4 °C. After being rinsed with PBS, corresponding fluorescent secondary antibodies were applied to the slices for 1 h. The slices were stained with DAPI, and images were captured and analyzed using a fluorescence microscope.

### Western blot analysis

Proteins from intestinal tissues and cells were extracted using 2 × SDS buffer with a protease inhibitor cocktail (Sigma-Aldrich). Protein concentrations were determined using the BCA protein assay kit (Sigma-Aldrich). Each sample, containing 15‒30 μg of protein, was separated by 8‒15% SDS-PAGE and transferred onto PVDF membranes (Millipore). The membranes were then blocked in 5% non-fat milk in 1 × TBST for 1 h at room temperature. Subsequently, the membranes were incubated with primary antibodies at 4 °C overnight, followed by incubation with corresponding secondary antibodies at room temperature for 1 h. The protein bands were visualized by an ECL kit (ThermoFisher Scientific) and quantified with ImageJ software.

### Analysis of mRNA expression

The expression levels of specific mRNAs were quantified by RT-qPCR with SYBR Green according to the manufacturer’s instructions and were normalized to *β-actin* mRNA levels. The sequences of the primers are listed in Table S2.

### Assessment of serum biochemical parameters

Serum biochemical parameters, including alanine aminotransferase (ALT), aspartate aminotransferase (AST), serum creatinine (SCr), blood urea nitrogen (BUN), amylase (AMS), and lipase (LPS), were detected using a Synchron CX7 autoanalyzer (Beckman Coulter, Brea, CA, USA).

### Isolation and culture of PPMs

PPMs were isolated from mice as previously described [29]. Briefly, mice aged 7–12 weeks were administered 3 mL of sterile thioglycollate broth (3%) intraperitoneally to induce peritoneal macrophages. Three days after the injection, mice were sacrificed by cervical dislocation and immersed in 75% ethanol. PPMs were obtained by rinsing the peritoneal cavity with 10 mL of RPMI medium 1640. Following washes, the cells were re-suspended in RPMI medium 1640 supplemented with 10% fetal bovine serum. PPMs (10^6^ cells per well) were seeded in 12-well plates and stimulated with rPrdx1 for hours as designed.

### Cell immunofluorescence

Cell immunofluorescence staining was performed according to a previously published method with several modifications [38]. PPMs were washed twice with PBS, followed by fixation in 4% paraformaldehyde for 10 min and permeabilization with 0.1% Triton X-100 for 15 min. The cells were washed 3 times with PBS and blocked with 5% BSA for 1 h. Then the cells were incubated with primary antibodies overnight at 4 °C. After the incubation period, the cells were washed 3 times with PBS and incubated with Alexa-conjugated secondary antibodies. Nuclei were stained with DAPI for 2 min. Cells were imaged using a fluorescence microscope (N2-DM4B, Leica, Illinois, USA) or a confocal microscope (SP8-DMIL, Leica).

### Extraction of proteins from cell-free supernatants

The cell-free supernatants were collected and centrifuged at 4 °C for 5 min at 12,000 × g. Following centrifugation, 700 μL of the upper liquid was mixed with 700 μL of methanol and 175 μL of trichloromethane by vortex. After another centrifugation at 12,000 × g for 5 min at 4 °C, the liquid separated into three layers. The upper liquid was removed, leaving the middle white thin film layer (protein) and the lower liquid. Subsequently, 700 μL of methanol was added and thoroughly mixed. After a final centrifugation at 12,000 × g for 5 min at 4 °C, the precipitates at the bottom were identified as the proteins. The supernatant was discarded, and an appropriate amount of 2 × SDS loading buffer was added for subsequent western blot analysis.

### Isolation of the cytosolic fraction

Subcellular fractionation of PPMs was performed following a digitonin-based fractionation method as previously described, with several modifications [29, 39]. Briefly, 6 × 10^6^ PPMs from WT mice were stimulated with 25 nM rPrdx1 for 3, 6 or 12 h. After treatment, the cells were washed 4 times with sterile cold PBS. The cells were then treated with 300 μL of 0.005% digitonin extraction buffer for 10 min on ice, and the supernatant containing the cytosol was collected by centrifugation. The residual cell fractions containing cell membranes, organelles and nuclei were boiled with 60 μL of 2 × SDS loading buffer and further subjected to immunoblot analysis with antibodies against Rab7, Lamp1, and β-actin to confirm the purity of the cytosolic fraction.

### Mitochondrial reactive oxygen species analysis

PPMs were harvested and washed twice with sterile PBS. The cells were then incubated with 5 μM MitoSox^TM^ Red (#M36008, ThermoFisher Scientific) for 30 min at 37 °C. After washing with PBS twice, the cells were collected by centrifugation. Then, the cells were resuspended in 200 μL of PBS and subjected to analysis of the average fluorescence intensity by flow cytometry, which was performed on a Cytek Dxp Athena flow cytometer. The staining data were analyzed using FlowJo_V10 software.

### Intracellular K^+^ analysis

PPMs were harvested and washed twice with sterile PBS. Then, 1 × 10^6^ PPMs were resuspended in 200 µL of deionized water. The cells were further frozen and thawed 3 times to enhance lysis. Then, the supernatant was collected by centrifugation and subjected to intracellular K^+^ measurement by an ion-selective electrode (AU680 Analyzer, Beckman Coulter, Inc., California, USA).

### Statistical analysis

Data are presented as mean ± SD from at least three independent experiments. Statistical analysis was performed using SPSS 22.0 software. Non-paired Student’s *t* test was used for comparisons between two groups, and multiple groups were compared using one-way analysis of variance (ANOVA) with a *post hoc* Bonferroni test. Linear correlations were analyzed using the Pearson correlation coefficient. The Kaplan–Meier method was used to analyze survival data, and the log-rank (Mantel–Cox) test was used to assess the differences in survival between the groups. The value of *p* < 0.05 was considered statistically significant.

## Supplementary information

Supplementary information

Supplemental Material

## Data Availability

All data generated during this study are available upon reasonable request to the corresponding author. RNA-sequencing data was acquired from Gene Expression Omnibus (GEO) datasets under accession number GSE117993.
