# Supplementary material for: Peroxiredoxin 1 promotes intestinal inflammation by activating the NLRP3 inflammasome in macrophages through lysosomal disruption in Crohn’s disease
Source: Cell Death Dis. 2025 Jul 26;16(1):565. doi: 10.1038/s41419-025-07898-1 (PMC12297276; doi:10.1038/s41419-025-07898-1)
Supplement: Supplementary file 1 — Supplementary information [file 41419_2025_7898_MOESM1_ESM.docx]

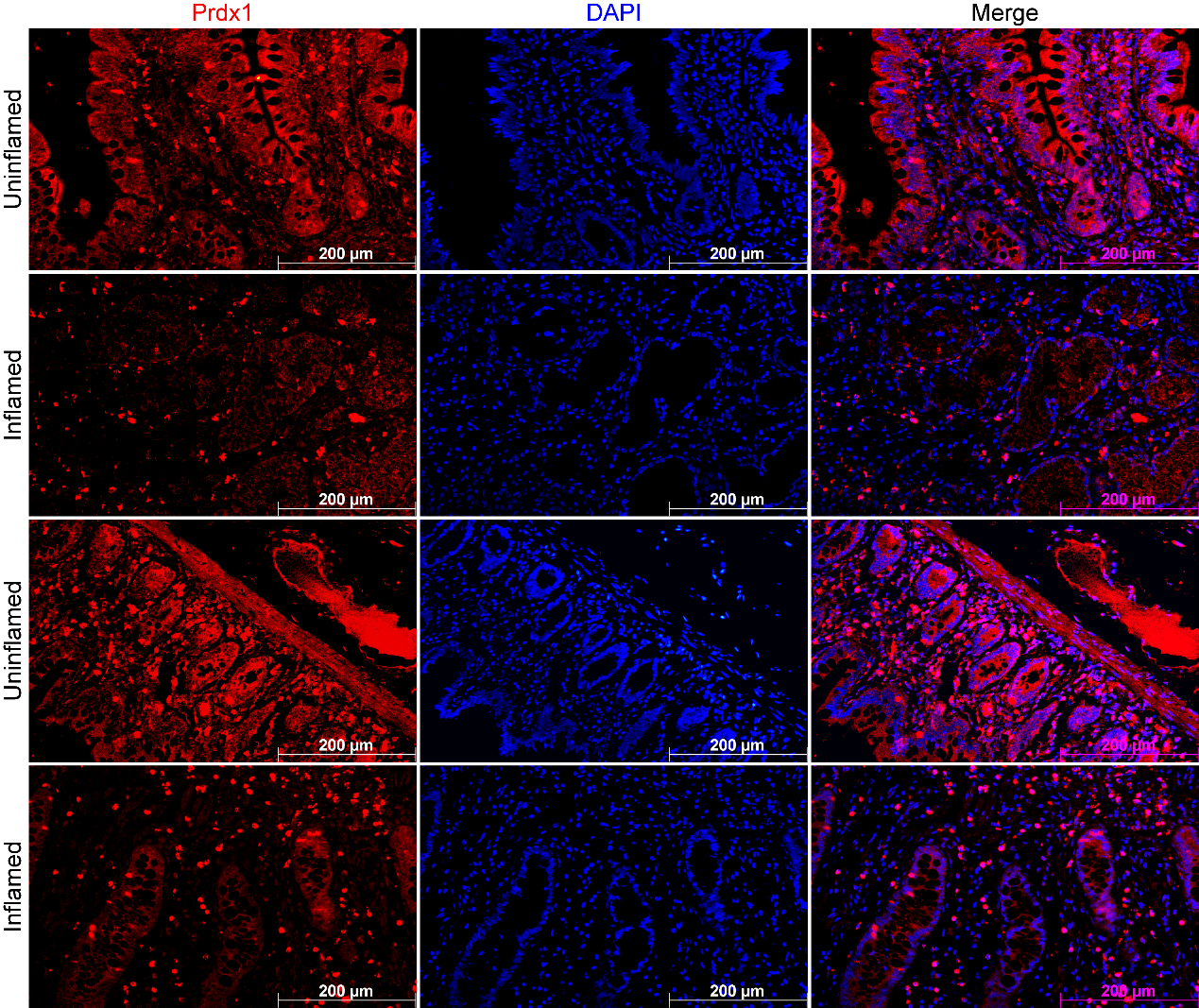


**Supplementary Fig. 1 The expression of Prdx1 was reduced in the inflamed tissues of CD patients.** Immunofluorescence staining for Prdx1 in the uninflamed and inflamed regions of the ileum/colon from the same CD patients. Typical images are shown. Scale bar, 200 μm.

**
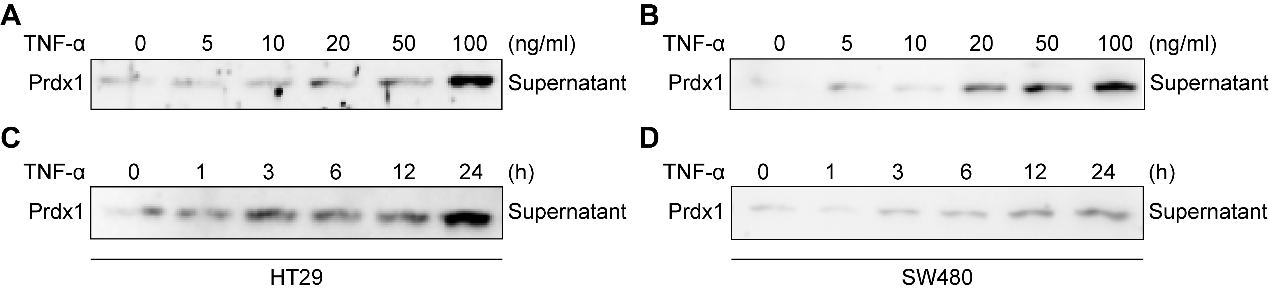
**

**Supplementary Fig. 2 Intestinal epithelial cells released Prdx1 into the extracellular space after injury. A, B** HT29 and SW480 cells were exposed to different doses of TNF-α (5, 10, 20, 50, and 100 ng/mL) for 24 hours (h). Conditioned medium was collected and analyzed for extracellular Prdx1 by western blot. **C, D** HT29 and SW480 cells were incubated with 100 ng/mL TNF-α for 1, 3, 6, 12, or 24 h. Conditioned medium was harvested and then subjected to western blot analysis of Prdx1. Results presented were repeated at least three independent experiments for each experimental condition.


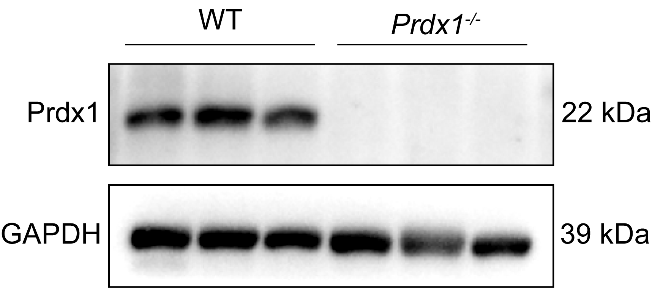
**Supplementary Fig. 3 *Prdx1* knockout (*Prdx1^–/–^*) efficiency was measured by western blot.** Prdx1 protein levels in colon tissues from *Prdx1^–/–^* mice and their littermates were measured by western blot. Results presented were repeated at least three independent experiments for each experimental condition.


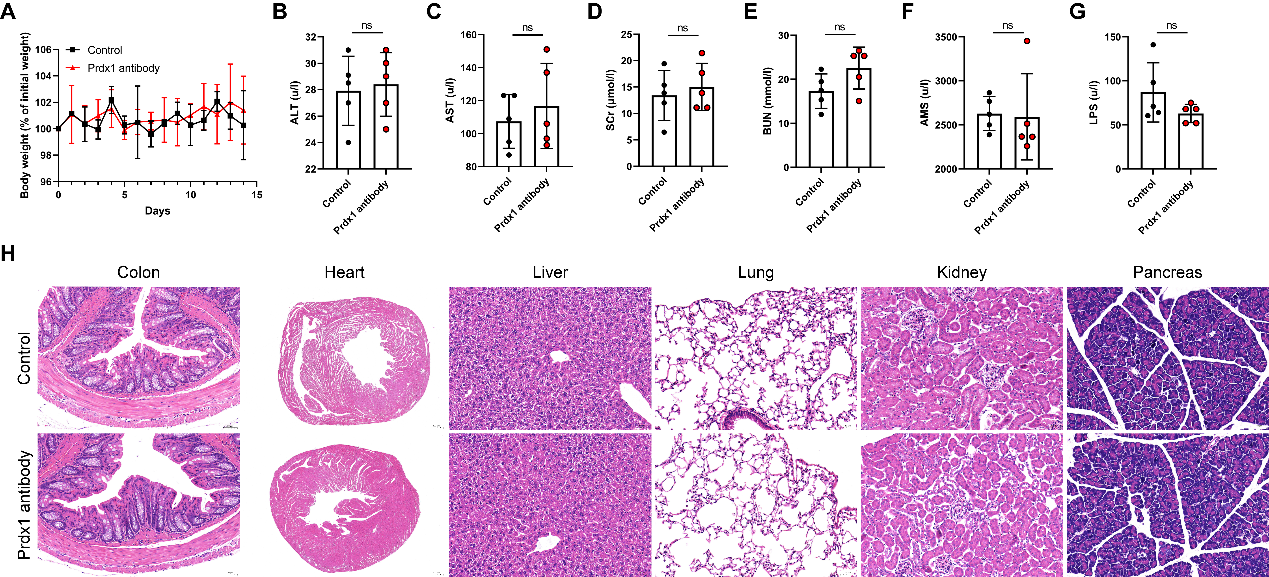


**Supplementary Fig. 4 Prdx1-neutralizing antibody exhibited a favorable safety profile.** Wild-type mice received intraperitoneal injection of a Prdx1-neutralizing antibody (150 μg/mouse) on Days 1, 3, and 5 and were monitored for 14 days (*n* = 5 mice per group). **A** Body weight of the mice was monitored daily and the data was plotted as a percentage of the initial body weight on Day 0. **B–G** Serum levels of biochemical parameters were measured in the indicated mouse groups, including alanine aminotransferase (ALT), aspartate aminotransferase (AST), serum creatinine (SCr), blood urea nitrogen (BUN), amylase (AMS), and lipase (LPS). **H** Representative H&E-stained sections of major organs from the indicated mouse groups. Scale bar, 50 μm (colon, liver, lung, kidney, pancreas) or 500 μm (heart). Data are expressed as mean ± SD; ^ns^*P* > 0.05 by non-paired Student’s *t* test. Results presented were repeated at least three independent experiments for each experimental condition.


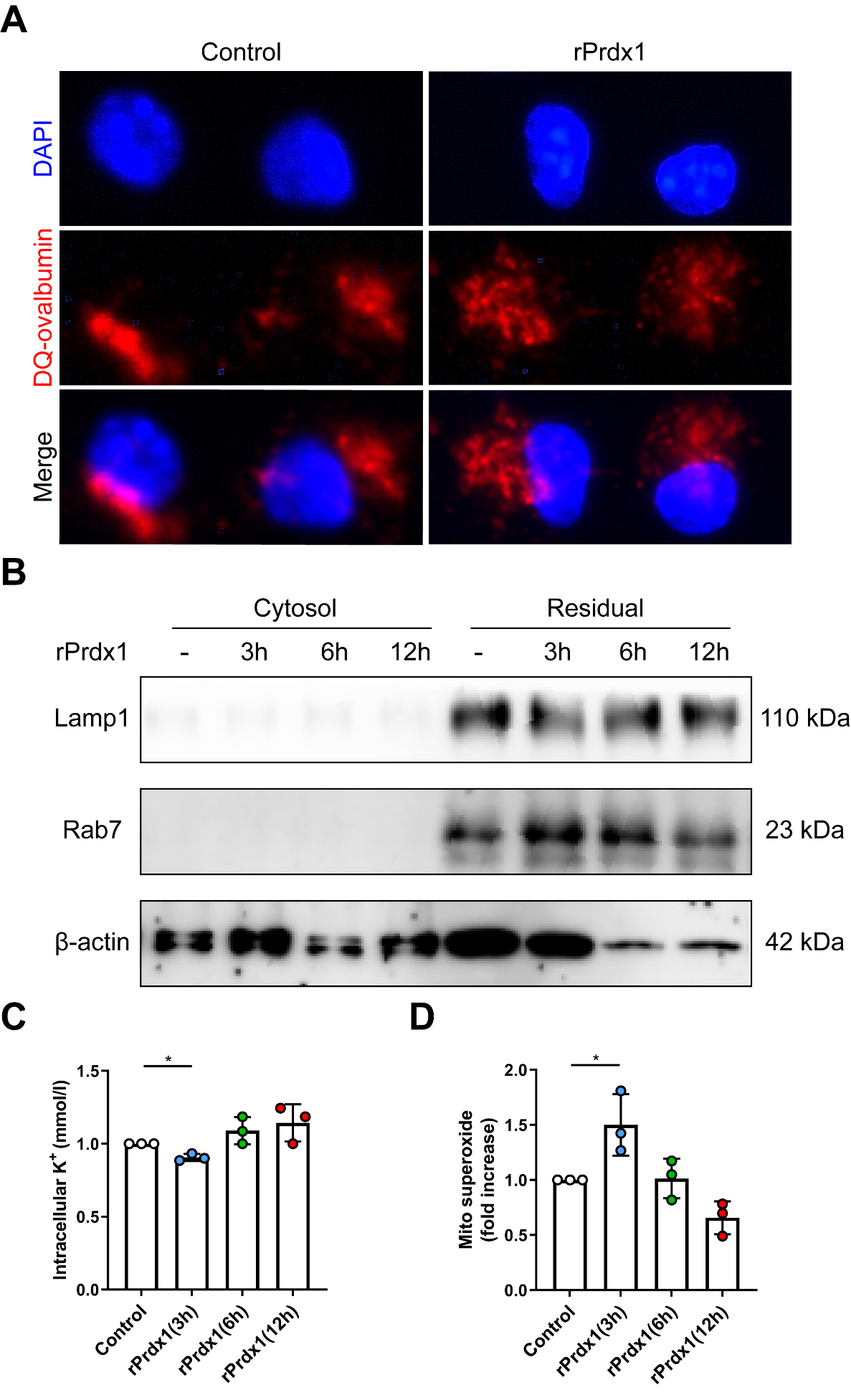
**Supplementary Fig. 5 Prdx1 activated the NLRP3 inflammasome via lysosomal disruption, independent of K^+^ efflux and mitochondrial reactive oxygen species (mtROS) production. A** Primary peritoneal macrophages (PPMs) were preincubated with DQ ovalbumin and then stimulated with 25 nM recombinant peroxiredoxin 1 (rPrdx1) for 12 hours (h). Immunofluorescence staining for DQ ovalbumin was conducted, and typical images are shown. **B** PPMs were stimulated with rPrdx1 (25 nM) for 3, 6, or 12 h. Then the cells were collected, and the cytosol and residual cell fraction were isolated using digitonin. The levels of Lamp1, Rab7, and β-actin in both the cytosolic and residual cell fraction were measured by western blot. **C, D** PPMs were stimulated with rPrdx1 (25 nM) for 3, 6, or 12 h. (**C**) The intracellular levels of K^+^ were quantified by an ion-selective electrode. (**D**) The mtROS levels in PPMs were determined using mitoSOX Red, and fluorescence intensity was analyzed by flow cytometry. Data are expressed as mean ± SD; ^*^*P* < 0.05 by non-paired Student’s *t* test. Results presented were repeated at least three independent experiments for each experimental condition.

**Supplementary tables**

| **Antibodies** | **Company** | **Catalog** | **Application** |
| --- | --- | --- | --- |
| Anti-rabbit peroxiredoxin 1 | Abcam, Cambridge, UK | ab15571 | WB/IF |
| Anti-rabbit F4/80 | Abcam, Cambridge, UK | ab300421 | IF |
| Anti-mouse NLRP3 | Adipogen, California, USA | AG-20B-0014 | WB |
| Anti-rabbit IL-1β | Santa Cruz, Texas, USA | sc-7884 | WB |
| Anti-mouse caspase-1 | Santa Cruz, Texas, USA | sc-56036 | WB |
| Anti-rabbit cathepsin D | Abcam, Cambridge, UK | ab75852 | WB |
| Anti-mouse Lamp2 | Santa Cruz, Texas, USA | sc18822 | IF |
| Anti-rabbit Rab7 | Cell Signaling Technology, Colorado, USA | 9367S | WB |
| Anti-mouse Lamp1 | ThermoFisher Scientific, Massachusetts, USA | 14-1071-85 | WB |
| Anti-mouse GAPDH | Sigma‒Aldrich, Darmstadt, Germany | G9295 | WB |
| Anti-mouse β-actin | Sigma‒Aldrich, Darmstadt, Germany | A2547 | WB |

**Supplementary Table 1. Antibodies and manufacturers**

**Supplementary Table 2. Sequences of primers**

| **Name** | **Forward (5' to 3')** | **Reverse (5' to 3')** |
| --- | --- | --- |
| *Prdx1* | GCCGCTCTGTGGATGAGATTA | AGCTGGACACACTTCACCAT |
| *IL-1β* | CTGGTGTGTGACGTTCCCAT | TCGTTGCTTGGTTCTCCTTGT |
| *IL-6* | ACCAAGAGATAAGCTGGAGTCAC | TAACGCACTAGGTTTGCCGA |
| *TNF-α* | CACCACGCTCTTCTGTCTACT | AACTGATGAGAGGGAGGCCAT |
| *β-actin* | CACTGTCGAGTCGCGTCC | TCATCCATGGCGAACTGGTG |

Prdx1, peroxiredoxin 1; IL, interleukin; TNF-α, tumor necrosis factor alpha.
