# Supplementary figures and images for: Peroxiredoxin 1 promotes intestinal inflammation by activating the NLRP3 inflammasome in macrophages through lysosomal disruption in Crohn’s disease
Source: Cell Death Dis. 2025 Jul 26;16(1):565. doi: 10.1038/s41419-025-07898-1 (PMC12297276; doi:10.1038/s41419-025-07898-1)

Fig. 1I

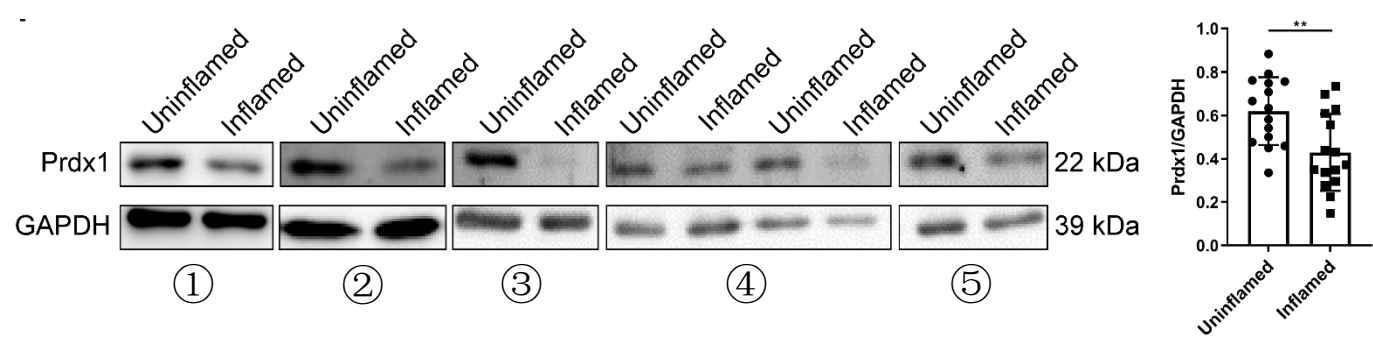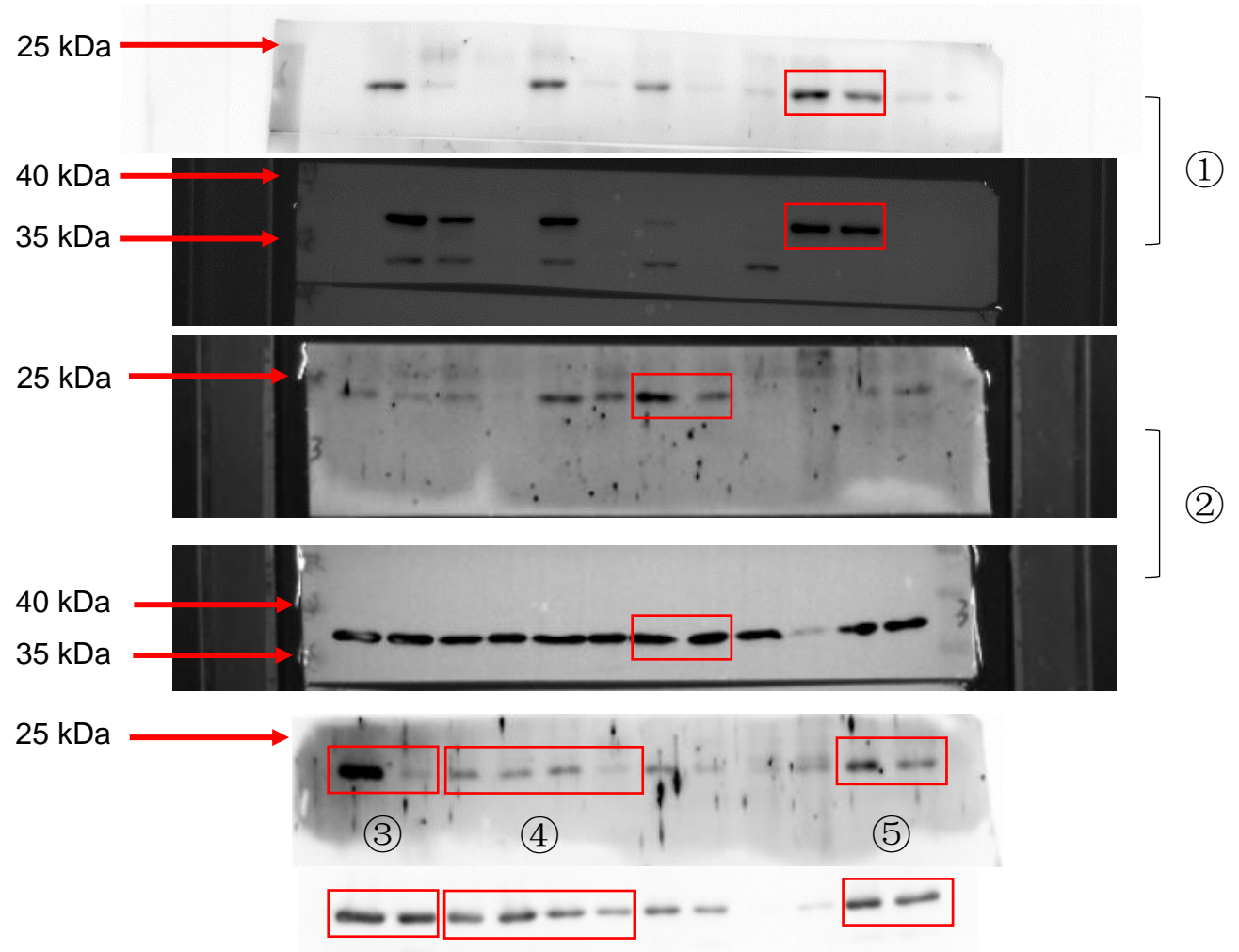

Fig. 2C

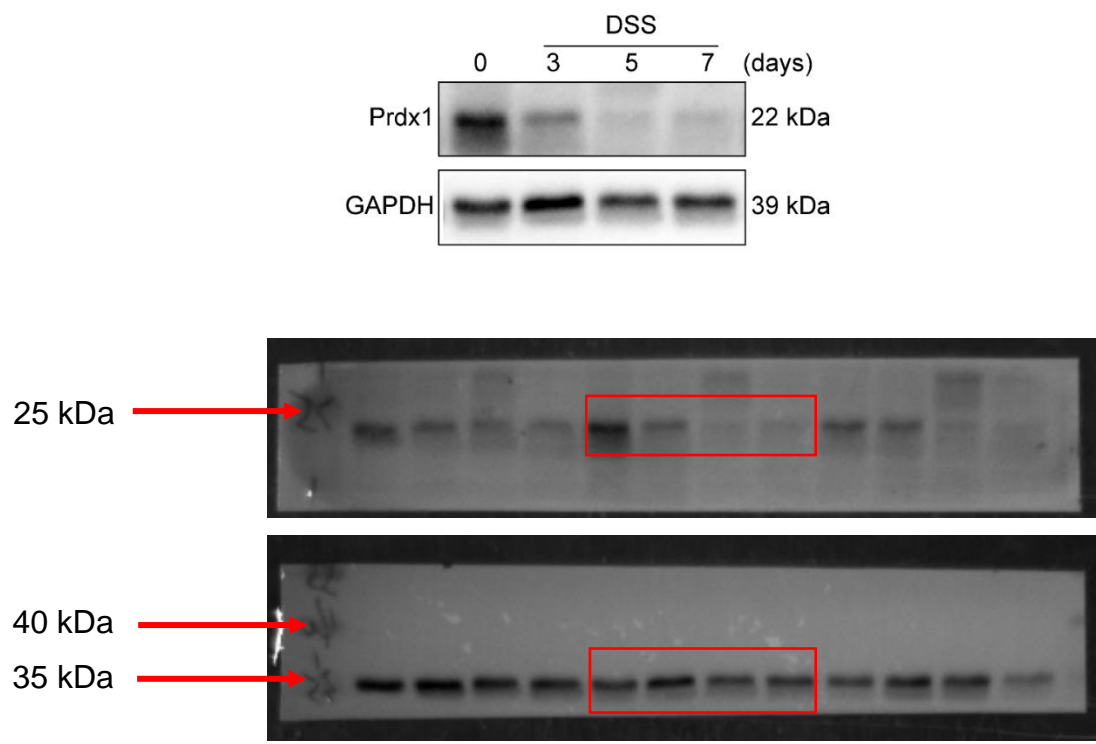

Fig. 7A

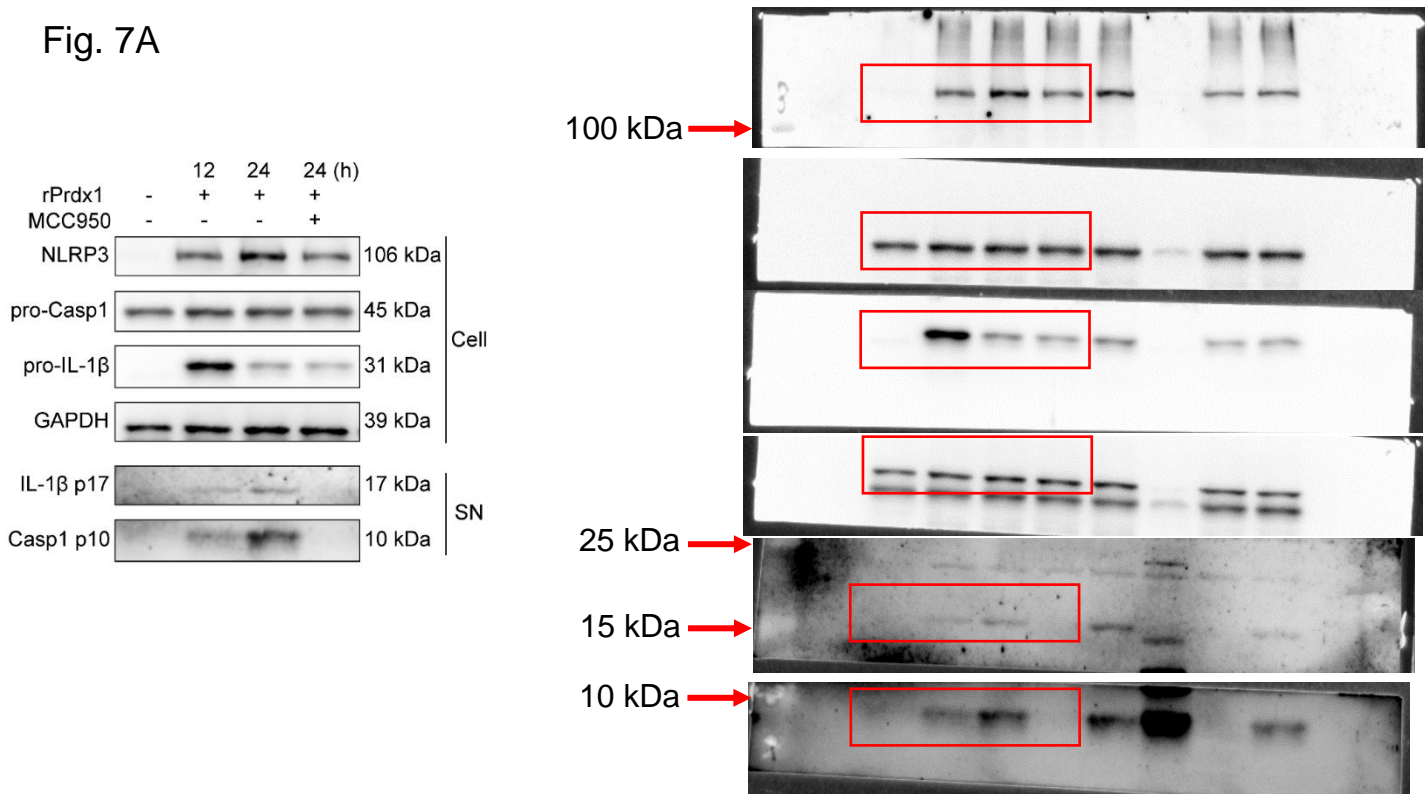

Fig. 7F

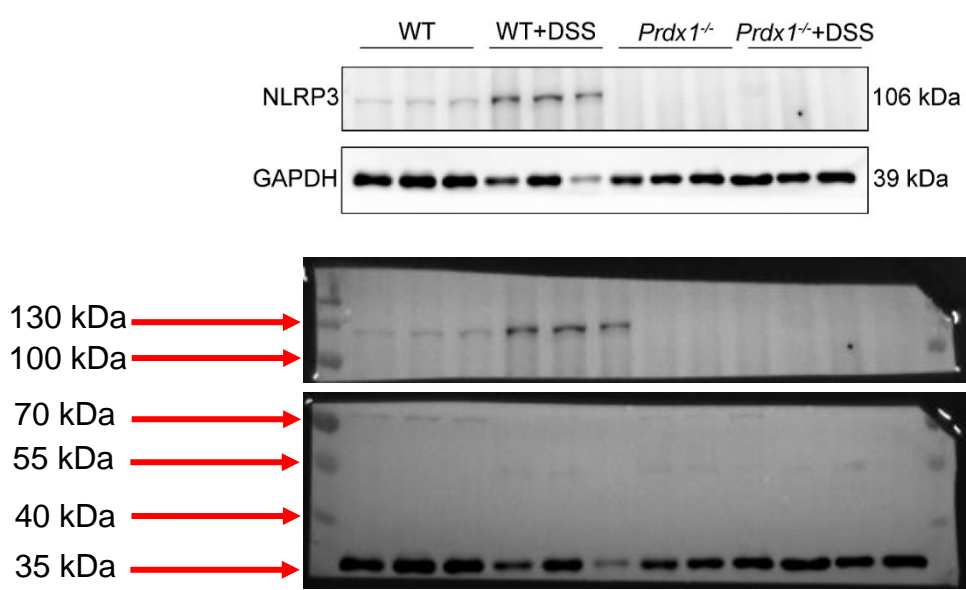

Fig. 7I

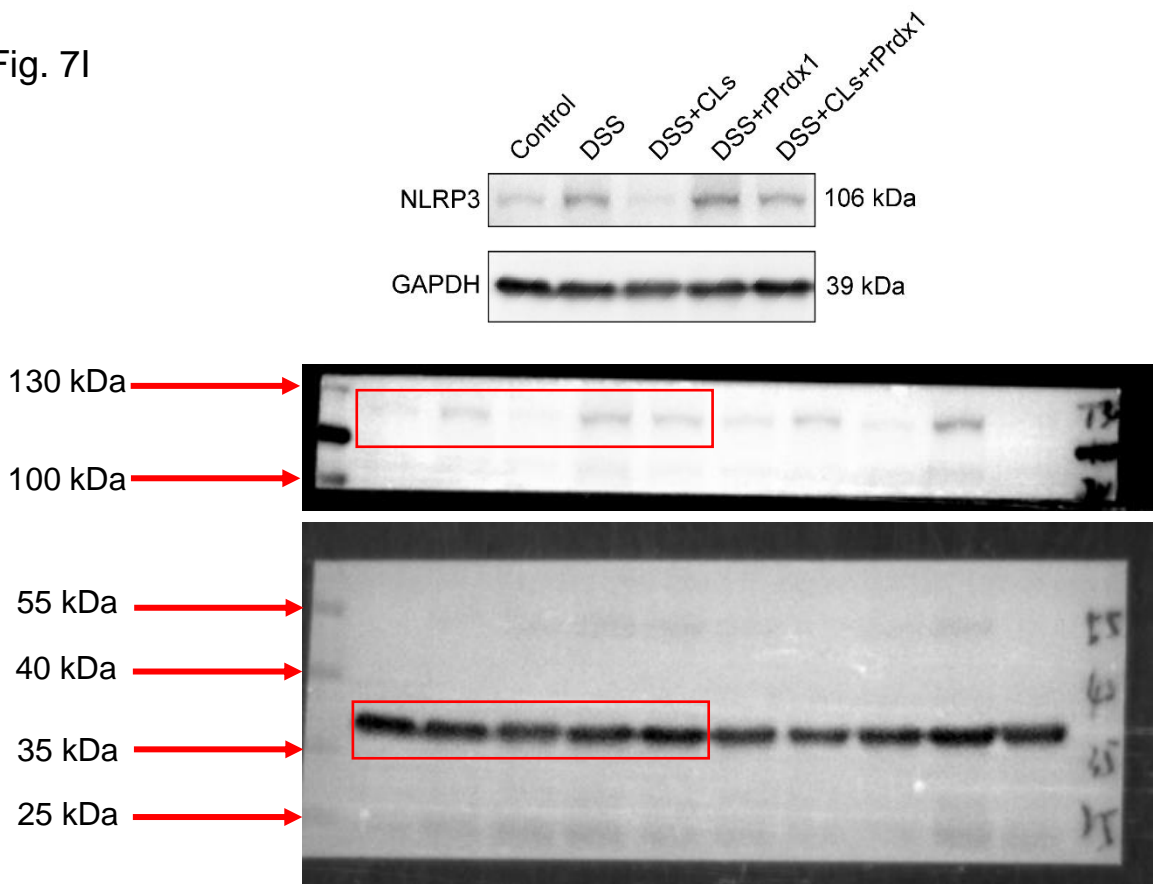

Fig. 8C

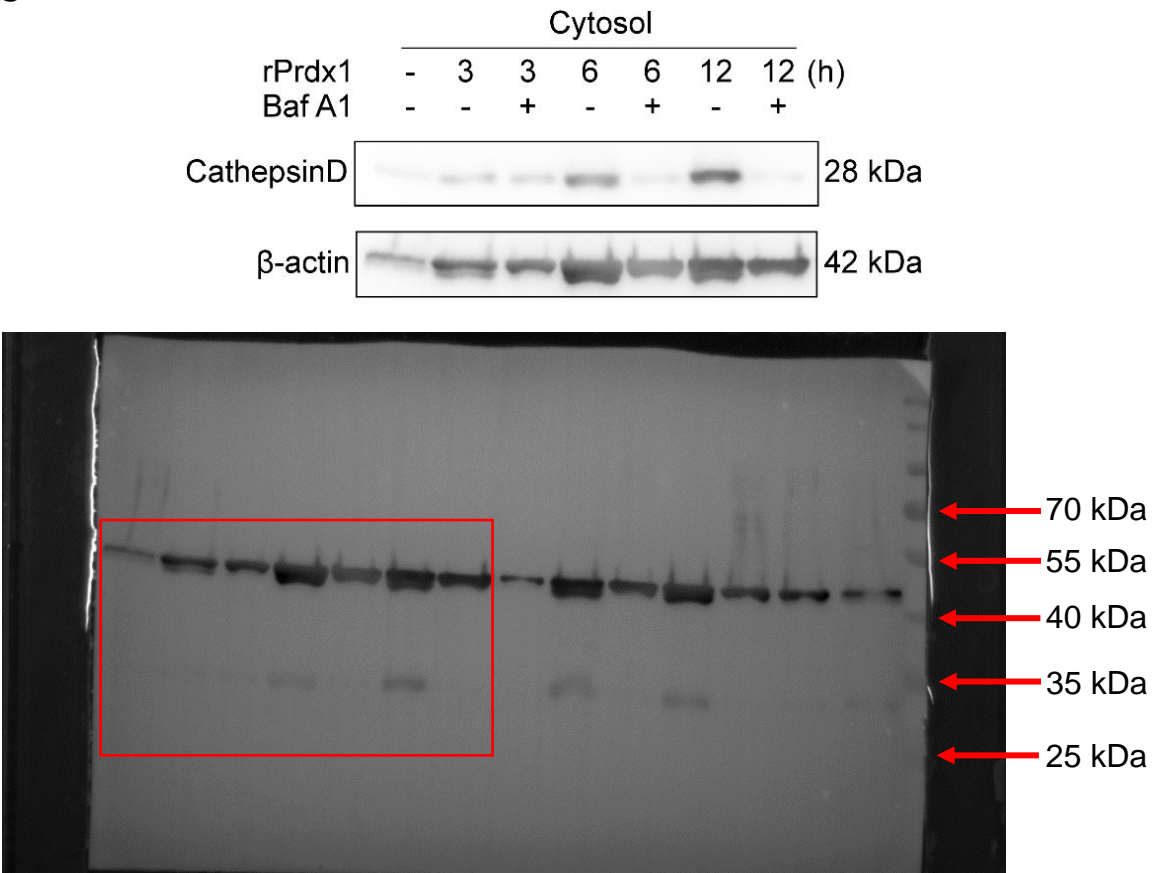

Fig. 9E

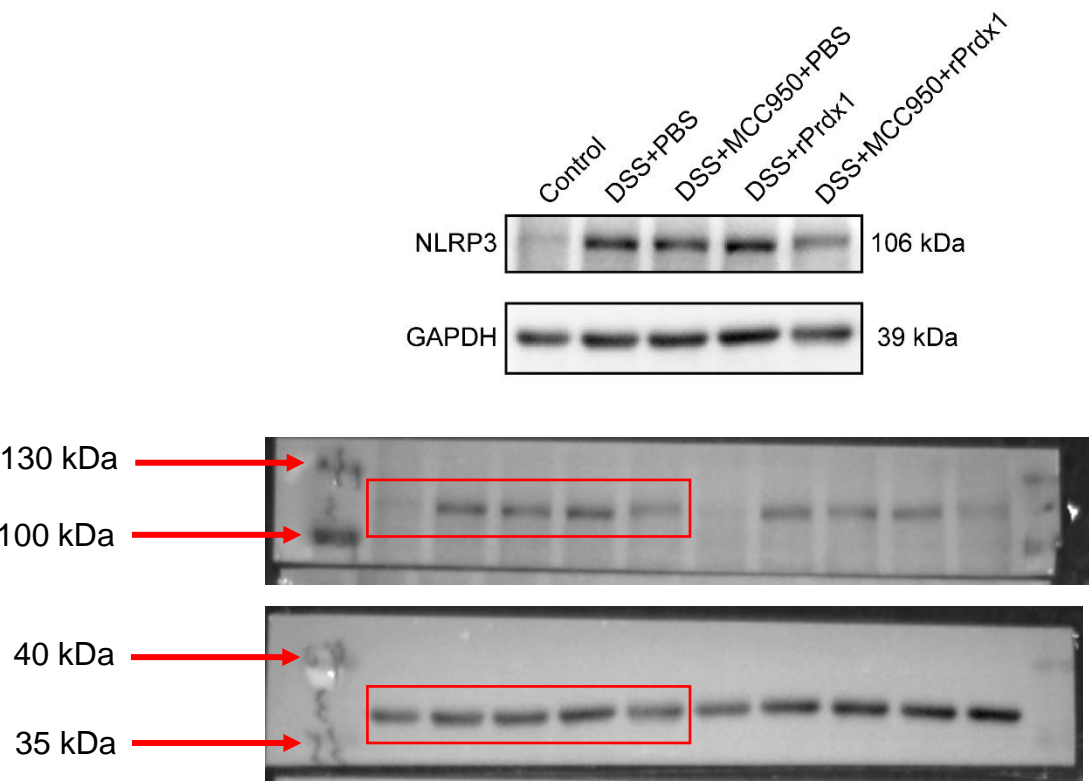

Supplementary Fig. 2

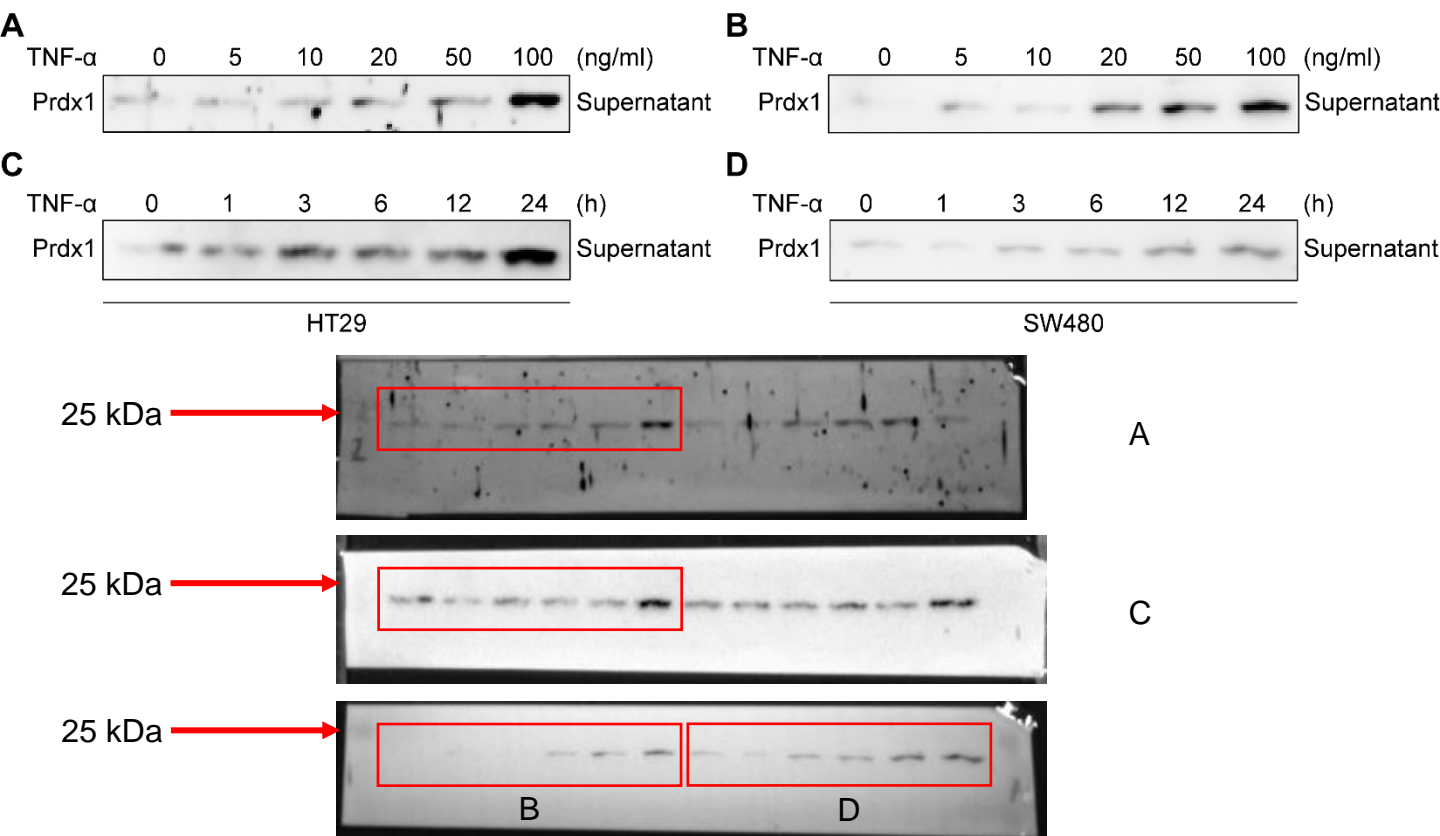

Supplementary Fig. 3

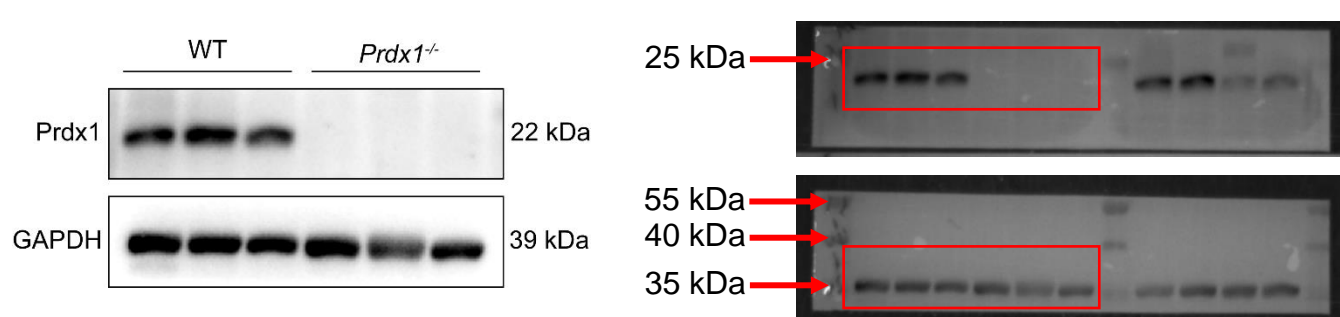

Supplementary Fig. 4B

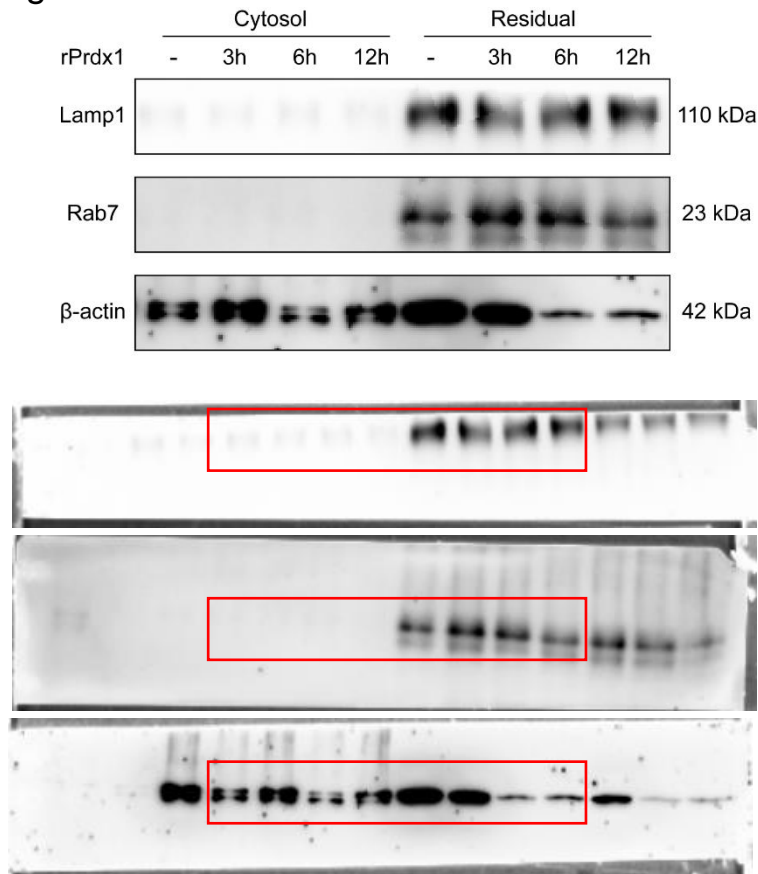

Supplement: Supplementary file 2 — Supplemental Material [file 41419_2025_7898_MOESM2_ESM.pdf]
